# Decision-Making and Environmental Implications under Cap-and-Trade and Take-Back Regulations

**DOI:** 10.3390/ijerph15040678

**Published:** 2018-04-04

**Authors:** Yuyu Chen, Bangyi Li, Qingguo Bai, Zhi Liu

**Affiliations:** 1 College of Economics and Management, Nanjing University of Aeronautics and Astronautics, Nanjing 211106, China; chenyuyu@nuaa.edu.cn (Y.C.); libangyi@263.net (B.L.); 2 Institute of Operations Research, School of Management, Qufu Normal University, Rizhao 276826, China; 3 College of Management Engineering, Anhui Polytechnic University, Wuhu 241000, China

**Keywords:** production and collection decision, remanufacturing, cap-and-trade regulation, take-back regulation, environmental implication

## Abstract

To reduce carbon emissions during production and realize the recycling of resources, the government has promulgated carbon cap-and-trade regulation and take-back regulation separately. This paper firstly analyses the manufacturing, remanufacturing and collection decisions of a monopoly manufacturer under cap-and-trade regulation and take-back regulation conditions, and then explores the environmental impact (i.e., carbon emissions) of both carbon regulation and more stringent take-back regulation. Finally, numerical examples are provided to illustrate the theoretical results. The results indicate that it will do good for the environment once the cap-and-trade regulation is carried out. We also conclude that government’s supervision of carbon trading price plays an important role in reducing the environmental impact. Furthermore, unexpectedly, we prove that if emissions intensity of a remanufactured (vis-á-vis new) product is sufficiently high, the improvement of collection and remanufacturing targets might lead to the deterioration of environment.

## 1. Introduction

Resource recycling and carbon emissions reduction have become hot topics. With the progress of technology and the improvement of living standards, the amount of waste electrical and electronic equipment (WEEE) has increased dramatically. According to statistics, the total amount of global WEEE was 41,800 thousand tons in 2017, and is expected to reach 50 million tons in 2018 [1]. On the other hand, with the increase of global temperature, the desertification of land and the frequency of extreme weather occurrence are both increasing. The harm of greenhouse effect is attracting more and more attention all over the world. Therefore, it is imperative for enterprises to realize the recycling of products at their end of life and reduce carbon emissions during production [2]. The government is responsible for the supervision of enterprises, on the one hand, the government promulgates carbon cap-and-trade regulation to regulate the carbon emissions during production [3], on the other hand, the government enacts take-back regulation by regulating the collection and remanufacturing targets to reduce the impact of WEEE landfill on the environment [4].

WEEE contains a large amount of renewable resources, which will adversely affect the environment unless they are properly disposed [5]. In order to promote the collection and utilization of resources, thereby reducing the amount of waste into landfills, take-back regulations have been enacted in many countries [6]. For example, according to the EU (European Union) WEEE Directive, the provisions of minimum recovery rate was 45% in 2016; the Japanese Specified Household Appliance Recycling Law (SHARL) sets a recycling rate between 50% and 60% [7]. China enacted the “Extended Producer Responsibility System Implementation Plan”, which clearly pointed out that the recovery and recycling target of WEEE is 40% by 2020 [8].

At present, there are two major types of WEEE take-back schemes: collective schemes and individual schemes. According to the collective scheme, which is implemented, for example, in the states in Minnesota, Vermont and Wisconsin in the United States, products from multiple manufacturers are co-mingled together and routed to recycling operations over remanufacturing. As another example, the companies Braun, Electrolux, HP (Hewlett-Packard), and Sony set up the European Recycling Platform (ERP) in response to the WEEE Directive. In individual schemes, the enterprise collects and recycles its brand-name products, such as Samsung and Cisco. From the view of take-back incentive and the system profits, manufacturer’s individual take-back model is optimal [9], hence, our research is based on the individual scheme.

General ways of WEEE processing include remanufacturing, reuse, recycling and disposal [10]. Among these measures, remanufacturing is gaining popularity as its effective maintenance of the intrinsic value of components and quality assurance of remanufactured products [11]. Remanufacturing has been recognized by various governments for the reason that it can effectively reduce the amount of waste and reuse old material [12]. Once an enterprise introduces the emerging mode of remanufacturing, it will develop into a hybrid manufacturing-remanufacturing system. Then, our first main goal is to explore how manufacturer make his manufacturing, remanufacturing and collection decisions according to different levels of take-back regulation.

On the other hand, in order to reduce carbon emissions during production, some countries and regions have enacted different laws and regulations, such as mandatory carbon emissions capacity [13], carbon tax [14] and carbon cap-and-trade [15]. For example, European Union (EU) launched the European Union Emission Trading System (EU-ETS) in 2005, California promulgated cap-and-trade regulations in 2011, and carbon tax regulations were adopted by Sweden and Finland in 1991 and 1990, respectively. At present, carbon tax has been gradually implemented in developed countries, and it has a considerable number of supporters. However, the carbon tax policy has not yet been applied in China due to the enthusiasm of Chinese enterprises for low-carbon technological innovation is not high, and the awareness of low-carbon environmental protection of the public is not strong.

Among different regulations of carbon emissions, the carbon cap-and-trade regulation is an effective market-based mitigation mechanism and has been implemented in many countries, such as Australia, Canada, Japan, and the USA [16]. In July 2003, the European Union promulgated the “EU’s Greenhouse Gas Emissions Trading Directive”. Inspired by the emission trading scheme in the European Union, China promulgated carbon cap-and-trade regulation in 2013. At present, seven carbon trading pilot programs have been established by five cities and two provinces of China [17]. Carbon cap-and-trade regulation can be viewed as an incentive policy of government for corporate emissions reductions, in which carbon emissions credit is considered as a commodity that allowed to be traded in the carbon trading market. If a firm’s actual amount of carbon emissions exceeds the carbon cap, he can buy carbon credits in the carbon trading market. Otherwise, the enterprise can sell the surplus carbon credits [18]. At present, 17 carbon trading systems have been established all over the word, covering 35 countries, 12 states (provinces), and seven cities. The transaction amount of these systems accounts for 40% of global GDP (Gross Domestic Product), and Global ETS turnover amount is up to 34 billion US dollars [19].

In the hybrid manufacturing-remanufacturing system under carbon emissions regulation, due to the heterogeneous substitution of new and remanufactured products, the manufacturer needs to make quantitative combinations of new products (higher margins, more carbon) and remanufactured products (less margins and less carbon) to minimize the carbon emissions and maximize profit simultaneously. Then decisions about carbon-related production should be formulated with the consideration of carbon emissions regulation. Hence, our second main goal is to research how collection and production decisions should be adjusted when considering carbon emissions, which is an important and strategic decision-making problem faced by the manufacturer.

The cap-and-trade regulation and take-back regulation promulgated by the government are intended to reduce the pollution of the environment. However, when making decisions, the enterprise will make optimal decisions from the perspective of its own interest, so it is necessary to study the environmental impact caused by the implementation of these two types of regulation. Therefore, our third and final goal is to examine whether/when the implementation of cap-and-trade regulation and take-back regulation can reduce environmental impact. More specifically, does it better to the environment when cap-and-trade regulation is considered? Whether the environmental impact will deteriorate with the more stringent collection and remanufacturing targets, which the government needs to pay more attention to.

The above goals can be translated into the following research questions: How production and collection decisions should be made by the manufacturer in the absence of (considering) carbon emissions? How does manufacturer adjust his decisions when considering take-back regulation (including only collection target, and collection, remanufacturing targets)? What is the impact of carbon regulation on the environment? Is more stringent take-back regulation necessarily conducive to environmental protection?

We are interested in all the above questions, and the main contributions of this paper are as follows. Firstly, we incorporate take-back regulation and cap-and-trade regulation into manufacturing, remanufacturing and collection decisions of a monopoly manufacturer. Secondly, we found that if emissions intensity of a remanufactured (vis-á-vis new) product is sufficiently high, the improvement of collection and remanufacturing targets might lead to the deterioration of environment. Finally, we provide it is friendlier to the environment when cap-and-trade regulation is considered. Furthermore, the result indicates that government’s supervision of carbon trading price plays an important role in reducing the environmental impact. 

It worth noting that this paper focuses on the decision-making analysis of a monopoly manufacturer, in other words, there exists a shortage of market competition and the manufacturer can act independently. Furthermore, the manufacturer adopts an individual scheme, which means he collects and recycles his brand-name products by himself. In addition, this paper is based on the assumption of complete information and determined demand.

The remainder of this paper is organized as follows: Section 2 is devoted to a review of the related literature. The problem and notation descriptions are presented in Section 3. The model formulation and decision analysis are presented in Section 4. Section 5 analyzes the environmental impact of cap-and-trade regulation and take-back regulation, and in order to analyze the effect of related parameters, such as collection and remanufacturing targets and cost saving, on the optimal manufacturing, remanufacturing and collection decisions, the total profits and carbon emissions, numerical examples are presented in Section 6. Finally, conclusions and potential future studies are discussed.

## 2. Literature Review

Many studies have focused on issues related to production decisions in a hybrid manufacturing-remanufacturing system, and our study is related to three streams of research. The first stream studies manufacturing and remanufacturing decisions in absence of regulation; the second stream investigates manufacturing and remanufacturing decisions under carbon emissions regulation; and the third stream researches manufacturing and remanufacturing decisions under take-back regulation:

(1) Manufacturing and remanufacturing decisions in absence of regulation

Remanufacturing not only can offer significant economic benefits for the enterprises but also can reduce environmental pollution. In order to gain economic and social responsibility, many companies actively involved in manufacturing, such as Huawei, Staples, IBM and Apple participate.

Currently, scholars have actively explored the field of remanufacturing and closed-loop supply chains. For example, assuming that there are no differences between new and remanufactured products, Ferrer et al. [20] investigated the co-pricing of new and remanufactured products with and without competition, respectively. Ferrer et al. [21] extended their study to the differential pricing of new and remanufactured products, and studied the production and pricing decisions of manufacturers. Based on the influence of customer bargaining, Zhu et al. [22] study centralized and decentralized decisions and supply chain coordination by analyzing the two-channel closed-loop supply chain. For strategic issues, Agrawal et al. [23] studied the relation between consumers’ perceived value of new and remanufactured products through a series of behavioral experiments. Subramanian et al. [24] separately analyzed the impact of component versatility on the OEM (Original Equipment Manufacturer) profit under OEM remanufacturing and third-party remanufacturing scenarios. Örsdemir et al. [25] explored manufacturers’ optimal product quality and quantity decisions when considering competition with an independent remanufacturer. Shi et al. [26] analyzed the effect of remanufacturing designs on market segmentation and trade-in prices. From the perspective of technological innovations, Galbreth et al. [27] illustrated how the rate of innovation affects reuse decisions.

As mentioned above, many studies has been done on manufacturing and remanufacturing decision-making. However, they did not take into account carbon emissions nor do they explore the impact of government guidance on collection and remanufacturing. In fact, government-led production decision-making is a hot topic for scholars.

(2) Manufacturing and remanufacturing decisions under carbon emissions regulations

Carbon emissions cannot only be used as an important indicator to evaluate the environment, but also will ultimately affect the profit of the manufacturer through carbon emissions trading. Therefore, there is growing interest in exploring a hybrid manufacturing-remanufacturing system considering carbon emissions. We will now briefly relate our paper to the related literature in the field of manufacturing and remanufacturing decisions considering carbon regulations.

Miao et al. [2] analyzed the optimal pricing, manufacturing and remanufacturing decisions of a manufacturer when considering trade-in, based on the carbon tax regulation and carbon cap-and-trade regulation, respectively. Shu et al. [28] explored a manufacturer’s manufacturing, remanufacturing and inventory decisions under cap-and-trade regulations. Based on the dual sourcing newsvendor framework, Bai et al. [29] proposed robust newsvendor models under both carbon tax and cap-and-trade regulations. Yenipazarli [30] studied the optimal production and pricing decisions of a manufacturer who participates in manufacturing and remanufacturing under carbon tax regulation and analyzes the impact of carbon tax and carbon cap-and-trade regulation on strategic decisions. Liu et al. [31] explored the impact of three carbon policies, including mandatory carbon emissions capacity, carbon cap-and-trade and carbon tax, on remanufacturing decisions with limited information on demand distribution. Wang et al. [32] characterized the optimal manufacturing and remanufacturing decisions of the manufacturer considering carbon cap-and-trade regulations and financial constraints. Chang et al. [33] established two-period models for independent and substitutable demands, and analyzed manufacturing and remanufacturing decisions of the monopolist manufacturer under cap-and-trade regulations. Fahimnia et al. [34] investigated the impact of carbon trading pricing on the closed-loop supply chain by comparison with the standard forward supply chain. Under carbon tax regulations, Yang et al. [35] incorporated quality variability into an acquisition and remanufacturing problem, where carbon footprints are considered during production. 

The government not only needs to supervise the carbon emissions of enterprises during production, but also needs to guide the collection and remanufacturing of WEEE. Although the existing literature also take into account environmental factors such as carbon emissions, they don’t analyze the selection of optimal solutions under take-back regulations.

(3) Manufacturing and remanufacturing decisions under take-back regulations

Production and collection decisions under take-back regulations have received considerable attention and scholars have studied the issue of remanufacturing under government intervention and guidance. Webster et al. [36] considered a two-period model with a manufacturer and a third-party remanufacturer, in which the manufacturer does not engage in remanufacturing, and analyzed the impact of take-back regulation on profits. Zhu et al. [37] constructed a two-stage model including a government and a manufacturer, and compared the remanufacturing sales subsidy and donation subsidy. To analyze the performance of the take-back regulation, Zhou et al. [38] considered a supply chain with a manufacturer and two competing recyclers. Esenduran et al. [7,39] analyzed the optimal decisions of the manufacturer under take-back regulations with and without the competition of the remanufacturer separately, and investigates the impact of take-back regulations on remanufacturing and the environment. Karakayali et al. [40] explores whether the remanufacturing target should be included in the recycling target. Esendurand et al. [41] hypothesizes that recycling of WEEE has net profits, which would lead to competition between recyclers and producers. They found that restraints on producer collection alone would lead to a reduction in landfill transfer and lower enthusiasm for product design. Atasu et al. [42] aimed at maximizing social welfare and studied the effective design of take-back regulations. Jacobs et al. [43] investigated the economic and environmental impacts of take-back regulations and studied how individual members should share responsibilities. From a stakeholders’ perspective, Atasu et al. [44] conducted a comparative study of tax policy and rate policy. Liu et al. [45] analyzed the trade-off problem between remanufacturing and recycling of WEEE and they studied the environmental impact using a LCA (Life Cycle Analysis) approach under the take-back regulation of China. Atasu et al. [46] studied how the recovery target of take-back legislation affected a firm’s product quality choice. Böni et al. [47] introduced a conformity assessment of WEEE take-back schemes in Switzerland. 

If the government regulates the collection and remanufacturing targets of WEEE considering carbon emissions, how will manufacturer adjust their decisions and how will cap-and-trade regulation and take-back regulation affect the environment? To address these issues, this paper incorporates take-back regulation and carbon cap-and-trade regulation into an enterprise’s manufacturing, remanufacturing and collection decisions. Based on the notion of maximization of manufacturer profit, the optimal production decision-making model is established, and the impacts of take-back regulations and carbon cap-and-trade regulations on the environment are discussed.

## 3. Problem Description and Notations

### 3.1. Problem Description

We assume there is a monopolist manufacturer (as in [7,30,32,40,45]), who produces and sells new products, and then collects and remanufactures them when these products reach their end of life. For simplification, we assume new and remanufactured products coexist, i.e., the manufacturer produces remanufactured products, even if there is no regulation [7]. Furthermore, he is able to collect its own products that consumers no longer want. In fact, many companies such as Huawei, Staples, Apple, and IBM are involved in such collection schemes. In addition, we assume that the product’s useful lifetime is one period and it can be remanufactured only once. These assumptions are quite commonly seen in real life [31,48].

Both the price and cost of a new product unit are higher than those of a remanufactured product unit, i.e., *p_n_* > *p_r_*, *c_n_* > *c_r_*. Consumers are heterogeneous with respect to their willingness to pay *θ* for a new product, where *θ* is uniformly distributed in the interval (0,1). Remanufactured products have the same functions as new ones, but are perceived inferior in quality by consumers [40]. Consequently, customers’ valuations for remanufactured products are lower than new products [23]. Therefore, consumers are willing to pay *δθ* for a remanufactured product, and δ∈(0,1) reflects the substitution degree of remanufactured products for new products [45]. 

The sales price of new and remanufactured products are $p_{n}$ and $p_{r}$ respectively, the utility functions of consumer for purchasing new and remanufactured products are $U_{n}=\theta -p_{n}$ and $U_{r}=\delta \theta -p_{r}$. Consumers are assumed to be rational decision-makers, hence, they purchase new products only if $U_{n}^{}>\max\{0,U_{r}^{}\}$, and they purchase remanufactured products only if $U_{r}^{}>\max\{0,U_{n}^{}\}$. Let $q_{n}$ and $q_{r}$ denote the demand for new and remanufactured products. We assume that a fixed market size is normalize to unity, then corresponding inverse functions are $p_{n}=1-q_{n}-\delta q_{r}$ and $p_{r}=\delta \left(1-q_{n}-q_{r}\right)$. For derivations of these expressions, we refer readers to [12,20].

We denote *e* as the carbon emissions by manufacturing a new product, and the carbon emissions of unit remanufactured product are less those of a unit of new product, hence the emissions generated to remanufacture unit product is αe [30], where α∈(0,1) denotes the emissions intensity of remanufacturing (vis-á-vis manufacturing) [49]. Therefore, the total carbon emissions during production are $E\left(q_{n},q_{r}\right)=eq_{n}+\alpha eq_{r}$.

To reduce carbon emissions during the production, the government has enacted carbon emissions regulations, such as carbon cap-and-trade regulations. Under cap-and-trade regulations, the unit trading price of carbon emissions and the carbon emissions cap are denoted by *ε* and K separately. If the actual total carbon emissions exceed the carbon cap, K, which is set by the government, the manufacturer can buy carbon permits at trading price *ε*. Conversely, the manufacturer can sell the extra carbon permits at the same price and obtain extra profit [3].

To reduce the impact of WEEE landfilling on the environment and realize the effective reuse of resources, the government has promulgated take-back regulation, such as WEEE Directive, in which it sets the collection target as 45% of new products put on the market, in addition to existing collection target, policy makers propose to set the remanufacturing target as 3% [39]. In this paper, the collection and remanufacturing targets are denoted by $\tau_{c}$ and $\tau_{r}$, respectively, where $\tau_{c}>\tau_{r}$.

**Figure 1:**
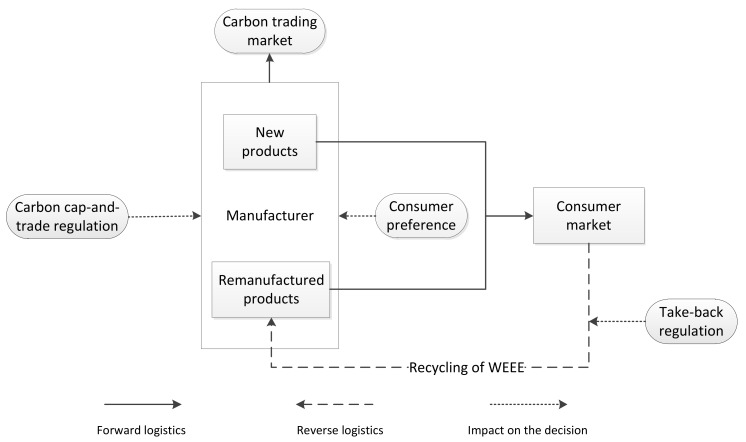
Hybrid manufacturing-remanufacturing system under cap-and-trade regulation and take-back regulation.

The decision processes can be seen in Figure 1, first, the government promulgates carbon cap-and-trade regulation and take-back regulation separately. The government should decide the carbon cap, K, and carbon trading price, *ε*, of the cap-and-trade regulation. Meanwhile, the government should determine the collection target, $\tau_{c}$, and remanufacturing target, $\tau_{r}$, of the take-back regulation. Under both cap-and-trade regulation and take-back regulation, considering the consumer preference, then the manufacturer makes manufacturing, remanufacturing and collection decisions.

In this paper, we consider a monopoly manufacturer who produces new and remanufactured products, where consumers are heterogeneous in their willingness-to-pay and value remanufactured products less than new products. Using a single-period steady state model, we examine two distinct cases: (1) without carbon emissions consideration; (2) considering carbon emissions. Under each case, we examine how the manufacturer optimizes his decisions under three different levels of take-back regulation (no take-back regulation, regulation only with collection target, regulation with additional remanufacturing target).

### 3.2. Notations

Table 1 summarizes the main parameters and notations used in this paper. The superscript “*” is added to the respective variables to represent their corresponding optimal values in the remainder of paper. *NR*, *R* and *RR* represent no take-back regulation, regulation only with collection target and regulation with both collection and remanufacture targets separately.

In addition, for convenience, we redefine $\gamma_{1}=1-c_{n}$, $\gamma_{2}=\delta -c_{c}-c_{r}=\delta -c_{c}-c_{n}+u$, where, $\gamma_{1}$ is net profit of unit new product and $\gamma_{2}$ is net profit of unit remanufactured product.

## 4. Model Formulation and Decision Analysis

This section formulates mathematical models for two different conditions: without carbon cap-and-trade regulation, and with carbon cap-and-trade regulation. Consequently, each condition can be divided into two sub-scenarios: without and with take-back regulation. For these four scenarios, the optimal manufacturing, remanufacturing and collection decisions are made for the manufacturer.

When the qualified manufacturer collects and remanufactures WEEE, the optimal strategy can be denoted as $X^{i,\;j}$, where, i={A,V,M} describes the relationship between collection amount and manufacturing quantity, while, j={A,V,M} describes the relationship between remanufacturing quantity and manufacturing quantity. “*A*” (All) represents that the manufacturer collects or remanufactures all WEEE, which indicates that the amount of collection/remanufacturing is equal to the quantity of manufacturing. Voluntary (“*V*”) represents that the manufacturer collects/remanufactures parts of WEEE voluntarily, i.e., $q_{n}^{*}>q_{c}^{*}>\tau_{c}q_{n}^{*}$ or $q_{n}^{*}>q_{r}^{*}>\tau_{r}q_{n}^{*}$. Mandatory (“*M*”) indicates a take-back regulation is mandatory, and the manufacturer collects/remanufactures according to some minimum target required by the government, i.e., $q_{c}^{*}=\tau_{c}q_{n}^{*}$ or $q_{r}^{*}=\tau_{r}q_{n}^{*}$. To distinguish solution 2 and solution 3, we add “*” after solution 2. The difference between them is that WEEE collected in solution 2 is not fully used for remanufacturing, i.e., $q_{c}^{*}>q_{r}^{*}$, while in solution 3, the manufacturer remanufactures all the WEEE which has been collected, i.e., $q_{c}^{*}=q_{r}^{*}$. The definitions of all solutions are shown in Table 2.

We select u and δ as the key parameters to analyze optimal strategies when not considering carbon emissions, because u is an important parameter reflecting the cost advantage of remanufacturing and δ reflects substitution degree of remanufactured products for new products. While u and α are selected as key parameters to analyze optimal strategies when considering carbon emissions, because α reflects emissions intensity of remanufacturing (vis-á-vis manufacturing). Bounds of u are listed in Table 3, where $t_{k}$ and $g_{k}$ (k∈{0,1,2,3,4}) are thresholds of u describing the characteristics of the optimal solutions without and with cap-and-trade regulation respectively, furthermore, we have $t_{1}>t_{2}>t_{3}>t_{4}>t_{0}>0$ and $g_{1}>g_{2}>g_{3}>g_{4}>g_{0}>0$.

### 4.1. Models Not Considering Cap-and-Trade Regulation

In absence of cap-and-trade regulation, the manufacturer engages in manufacturing and remanufacturing ignoring carbon emissions, the profit of the manufacturer is as follows:

(1) $$\begin{array}{c} \underset{\left\{q_{n},q_{r},q_{c}\right\}}{\max\;\Pi }=\left(p_{n}-c_{n}\right)q_{n}+\left(p_{r}-c_{n}+u\right)q_{r}-c_{c}q_{c}\\ s.t.\;\tau_{c}q_{n}\leq q_{c}\leq q_{n}\\ \;\;\;\tau_{r}q_{n}\leq q_{r}\leq q_{c} \end{array}$$

In the above profit function, the first two parts are sales revenue of new and remanufactured products and the last part is collection cost. Furthermore, the first constraint ensures that the collection amount of the manufacturer is at least as much as required by the government and cannot exceed the manufacturing quantity in the preceding period. While, the second constraint ensures that the remanufacturing quantity is at least as much as required by the government and cannot exceed the collection amount.

#### 4.1.1. The Model without Take-Back Regulation

We firstly study optimal decisions of the manufacturer without take-back regulations, i.e., $\tau_{c}=\tau_{r}=0$.

**Proposition** **1.** *In absence of take-back regulation, ∃* $t_{0}$ *and* $t_{1}$ *(see in Table 3) such that if* $t_{0}<u<t_{1}$*, the optimal solution of the manufacturer is NR^VV^; otherwise (i.e.,* $u\geq t_{1}$*), the optimal solution is NR^AA^.*

Proof. All proofs are provided in the Appendix A.

**Figure 2:**
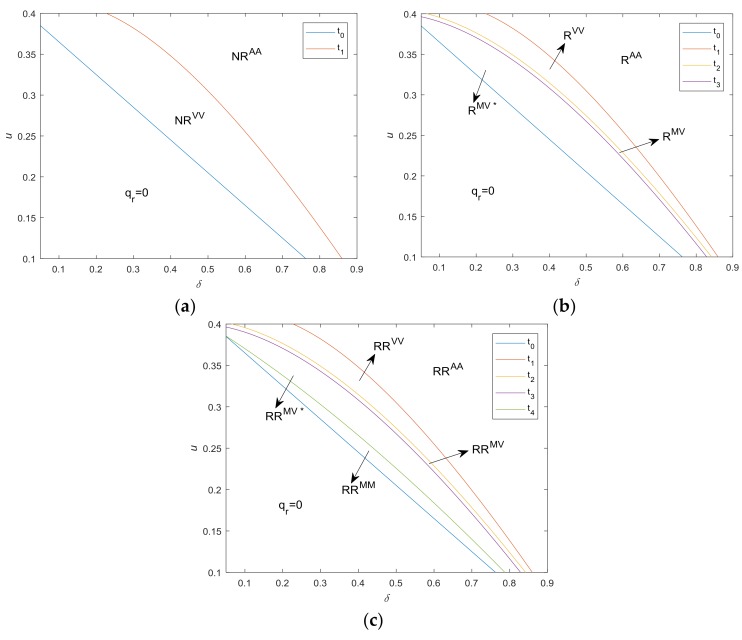
Optimal strategies in absence of cap-and-trade regulation. (**a**) no take-back regulation; (**b**) under take-back regulation with collection target; (**c**) under take-back regulation with both collection and remanufacturing targets. (Note, $c_{n}=0.4$, $c_{c}=0.05$, α=0.5, $\tau_{c}=0.6$, $\tau_{r}=0.2$).

Figure 2a illustrates the optimal strategies with respect to u and δ without take-back regulations. Proposition 1 and Figure 2a confirm us that, when both cost saving of remanufacturing, u, and consumer value discount for the remanufactured products, δ, are quite high, the manufacturer is more interested in remanufacturing, and collects and manufactures all available WEEE. Conversely, when $t_{0}<u<t_{1}$, although remanufacturing can be profitable, remanufactured products have negative impact on the sales of new products. In this case, in order to maximize the overall profit, the manufacturer only collects part of the WEEE.

**Corollary** **1.** *The manufacturer enters remanufacturing market only when* $\frac{\gamma_{2}}{\gamma_{1}}>\delta$.

$\gamma_{1}$ and $\gamma_{2}$ are the net profit of unit new and remanufactured products, respectively. Corollary 1 shows that only when the remanufacturing has a certain competitive advantage (i.e., $\frac{\gamma_{2}}{\gamma_{1}}>\delta$) will the manufacturer enter remanufacturing market.

#### 4.1.2. The Model with Take-Back Regulation

As described above, take-back regulations can be divided into two cases: (1) regulation only with collection targets; (2) regulation with both collection and remanufacturing targets. In this subsection, two situations with and without additional remanufacturing target are analyzed. 

Hereafter, we firstly study the optimal decision of the manufacturer under regulation only with collection targets, i.e., $\tau_{c}>0$ and $\tau_{r}=0$.

**Proposition** **2.** *Under regulation only with collection targets, the optimal solution of the manufacturer is characterized as follows: ∃* $t_{2}$ *and* $t_{3}$*, where* $t_{1}>t_{2}>t_{3}>t_{0}$*, such that (i) if* $t_{0}<u<t_{3}$, the optimal solution of the manufacturer is R^MV*^; (ii) if $t_{3}\leq u\leq t_{2}$*, the optimal solution is R^MV^; (iii) otherwise (i.e.,* $u>t_{2}$*), the optimal solution is the same as ones under no regulation.*

Figure 2b illustrates the optimal strategies with respect to u and δ under take-back regulations only with collection target. Proposition 2 has an interesting implication. When both cost saving of remanufacturing u and consumer value discount for the remanufactured products, δ, are relatively high, i.e., $u>t_{2}$, the manufacturer collects WEEE voluntarily and the amount is higher than the collection target required by the government, therefore the collection target is redundant, while as the u and δ decrease, the manufacturer is more inclined to produce new products, leading to a reduction in the reactivity to remanufacture WEEE. When $t_{0}<u<t_{2}$, take-back regulation with collection target is mandatory and manufacturer collects manufacturer according to collection target $\tau_{c}$. Furthermore, when $t_{2}<u<t_{3}$, the manufacturer remanufactures all the WEEE collected, i.e., $q_{c}^{*}=q_{r}^{*}$. With a further reduction of u and δ, the WEEE collected is not fully used for remanufacturing, i.e., $q_{c}^{*}>q_{r}^{*}$.

**Corollary** **2.** ∃ $\bar{\tau_{c1}}\;$ *and* $\bar{\tau_{c2}}\;$*, and* $\bar{\tau_{c1}}\;<\bar{\tau_{c2}}\;$*, (i) When* $\tau_{c}\leq \bar{\tau_{c1}}\;$*, the take-back regulation has no effect on optimal decisions; (ii) When* $\tau_{c}>\bar{\tau_{c1}}\;$*, take-back regulation is mandatory. Furthermore, if* $\bar{\tau_{c1}}\;<\tau_{c}<\bar{\tau_{c2}}\;$*, the optimal decision is R^MV^, otherwise, i.e.,* $\tau_{c}>\bar{\tau_{c2}}\;$, the optimal decision is R^MV*^. Where $\bar{\tau_{c1}}\;=\frac{\gamma_{2}-\gamma_{1}\delta }{\delta \left(\gamma_{1}-\gamma_{2}\right)}$ *and* $\bar{\tau_{c2}}\;=\frac{\delta \left(\gamma_{1}-\gamma_{2}-2c_{c}\right)+\sqrt{\delta^{2}{\left(\gamma_{1}-\gamma_{2}-2c_{c}\right)}^{2}-4c_{c}\delta \left(\gamma_{2}+c_{c}-\gamma_{1}\delta \right)}}{2c_{c}\delta }$.

Corollary 2 shows that when the collection target is very low, take-back regulations have no effect on the decision, and the manufacturer collects and remanufactures voluntarily. Otherwise, i.e., $\tau_{c}>\bar{\tau_{c1}}\;$, take-back regulation is mandatory. More specifically, when $\bar{\tau_{c1}}\;<\tau_{c}<\bar{\tau_{c2}}\;$, all the collected WEEE will be remanufactured, and when the collection target is very high, i.e., $\tau_{c}>\bar{\tau_{c2}}\;$, only parts of collected WEEE will be remanufactured, and the other will be disposed. In other words, although the manufacturer collects WEEE according to collection target, however he does not reprocess it properly. In this case, it is especially necessary for the government to formulate additional remanufacturing target. Consequently, we will study the optimal decisions of the manufacturer under take-back regulation with both collection and remanufacturing targets, i.e., $\tau_{c}>\tau_{r}>0$.

**Proposition** **3.** *Under regulations with both collection and remanufacturing targets,* ∃ $t_{4}$*, where* $t_{3}>t_{4}>t_{0}$*, such that (i) if* $t_{0}<u\leq t_{4}$*, the optimal solution is RR^MM^; (ii) otherwise (i.e.,* $u>t_{4}$*), the optimal strategies are the same as ones under regulation with collection target alone.*

Figure 2c illustrates the optimal strategies under take-back regulations with both collection and remanufacturing targets. Compared with Figure 2b, the only difference is that when $t_{0}<u\leq t_{4}$, the optimal strategy changes from RR^MV*^ to RR^MM^. Proposition 3 shows that under take-back regulations with additional remanufacturing target, when $u>t_{4}$, the optimal decision is the same as that under regulations only with collection targets. While when $t_{0}<u\leq t_{4}$, the remanufacturing target is mandatory, i.e., $q_{r}^{*}=\tau_{r}q_{n}^{*}$, which implies that the regulation with additional remanufacturing targets will play a role only when u and δ are relatively low.

**Corollary** **3.** ∃ $\bar{\tau_{r}}\;$*, when* $\tau_{r}>\bar{\tau_{r}}\;$*, the remanufacturing target is mandatory, and the optimal decision is RR^MM^. Otherwise, regulation with additional remanufacturing target has no effect on optimal decisions, and the manufacturer remanufactures voluntarily, where* $\bar{\tau_{r}}\;=\frac{\gamma_{2}-\gamma_{1}\delta +c_{c}\left(1+\delta \tau_{c}\right)}{\delta \left(\gamma_{1}-\gamma_{2}-c_{c}-c_{c}\tau_{c}\right)}$.

The optimal decisions of the manufacturer are shown in Table 4, and the optimal profits and carbon emissions are shown in Table 5.

### 4.2. Models Considering Cap-and-Trade Regulation

As mentioned above, under carbon cap-and-trade regulations, the manufacturer can buy or sell carbon credits at the same trading price in the carbon trading market according to its production scheme. On the other hand, the collection (remanufacturing) rate of the manufacturer is not less than the collection (remanufacturing) target required by the government. Therefore, a model that determines optimal manufacturing, remanufacturing and collection amount to maximize the total profit is formulated as follows:

(2) $$\begin{array}{c} \underset{\left\{q_{n},q_{r},q_{c}\right\}}{\max\;\Pi }=\left(p_{n}-c_{n}\right)q_{n}+\left(p_{r}-c_{r}\right)q_{r}-c_{c}q_{c}-\varepsilon (eq_{n}+\alpha eq_{r}-K)\\ s.t.\;\tau_{c}q_{n}\leq q_{c}\leq q_{n}\\ \;\;\;\tau_{r}q_{n}\leq q_{r}\leq q_{c} \end{array}$$

Different from the model not considering carbon emissions, the manufacturer can trade carbon credits, hence, the last part of profit function is the income (or cost) of carbon emissions trading under carbon cap-and-trade regulation. The explanation of constraints is the same as that without cap-and-trade regulations.

#### 4.2.1. The Model without Take-Back Regulation

In order to explore the impact of take-back regulation on the choice of optimal strategies, we firstly study the optimal decisions of the manufacturer without take-back regulations.

**Proposition** **4.** *In absence of take-back regulation,* ∃ $g_{1}$*(in* Table 3*) such that if* $g_{0}<u<g_{1}$*, the optimal solution of the manufacturer is NR^VV^; otherwise (i.e.,* $u\geq g_{1}$*), the optimal solution is NR^AA^.*

**Figure 3:**
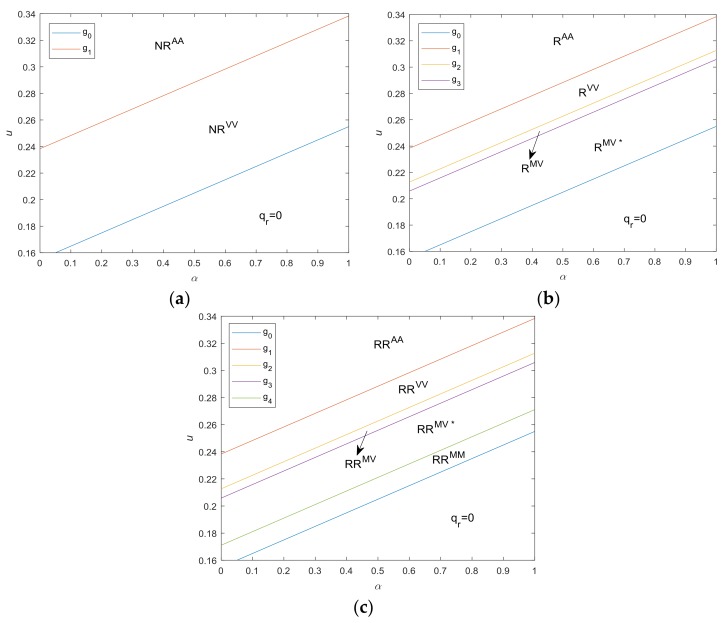
Optimal strategies under cap-and-trade regulation. (**a**) no take-back regulation; (**b**) under take-back regulation only with collection target; (**c**) under take-back regulation with both collection and remanufacturing targets. (Note, $c_{n}=0.4$, $c_{c}=0.05$, δ=0.5, e=0.5, ε=0.2, $\tau_{c}=0.6$, $\tau_{r}=0.2$).

Figure 3a illustrates the optimal strategies with respect to u and α without take-back regulations. Proposition 4 confirms us that, when the cost saving of remanufacturing, u, is high and the emissions intensity of remanufacturing, α, is small, the manufacturer is more interested in remanufacturing, and collects/remanufactures all available WEEE. Conversely, when $g_{0}<u<g_{1}$, although remanufacturing may be profitable, remanufactured products will have a negative impact on the sales of new products, implying a demand cannibalization. Therefore, in order to maximize the overall profit, the manufacturer only collects part of the WEEE.

**Corollary** **4.** *The manufacturer enters remanufacturing market only when* $\frac{\gamma_{2}-\alpha e\varepsilon }{\gamma_{1}-e\varepsilon }>\delta$.

$\gamma_{1}-e\varepsilon$ and $\gamma_{2}-\alpha e\varepsilon$ are net profits of unit new and remanufactured products considering carbon emissions. Corollary 1 shows that only when the remanufacturing has a certain competitive advantage (i.e., $\frac{\gamma_{2}-\alpha e\varepsilon }{\gamma_{1}-e\varepsilon }>\delta$), will the manufacturer enter the remanufacturing market.

#### 4.2.2. The Model with Take-Back Regulation

In this model, aside from focusing on carbon cap-and-trade regulations during production, the manufacturer should also pay attention to take-back regulations during the sales phase. Therefore, the manufacturer needs to adjust his production and collection strategies to maximize his profit. Similar to the model without cap-and-trade regulations, we also consider two cases: with and without additional remanufacturing target. Hereafter, we firstly study the optimal decisions of the manufacturer under regulation only with collection target, i.e., $\tau_{c}>0$ and $\tau_{r}=0$.

**Proposition** **5.** *Under regulations with collection targets, the optimal solution of the manufacturer is characterized as follows:* ∃ $g_{2}$ *and* $g_{3}$*, where* $g_{1}>g_{2}>g_{3}>g_{0}$*, such that (i) if* $g_{0}<u<g_{3}$, the optimal solution of the manufacturer is R^MV*^; (ii) if $g_{3}\leq u\leq g_{2}$*, the optimal solution is R^MV^; (iii) otherwise (i.e.,* $u>g_{2}$*), the optimal solution is the same as under no regulations.*

Figure 3b illustrates the optimal strategies with respect to u and α under take-back regulations only with collection targets. Proposition 2 has an interesting implication. When the cost savings of a remanufactured product, u, are high and emissions intensity of a remanufactured (vis-á-vis new) product, α, is low, i.e., $u>g_{2}$, the manufacturer collects WEEE voluntarily and the collection amount is more than the target required by the government, therefore the collection target is redundant. With the decrease of u and the increase of α, the manufacturer is inclined to produce new products, leading to a reduction in the reactivity to remanufacture WEEE. When $g_{0}<u<g_{2}$, take-back regulation with collection target is mandatory and manufacturer collects WEEE according to a collection target $\tau_{c}$. Furthermore, when $g_{2}<u<g_{3}$, the manufacturer remanufactures all the WEEE collected, i.e., $q_{c}^{*}=q_{r}^{*}$. With a further reduction of u and increase of α, only part of WEEE collected is used for remanufacturing, i.e., $q_{c}^{*}>q_{r}^{*}$.

**Corollary** **5.** ∃ $\hat{\tau_{c1}}$ *and* $\hat{\tau_{c2}}$*, and* $\hat{\tau_{c1}}<\hat{\tau_{c2}}$*. (i) When* $\tau_{c}\leq \hat{\tau_{c1}}$*, the take-back regulation has no effect optimal decisions. (ii) When* $\tau_{c}>\hat{\tau_{c1}}$*, take-back regulation is mandatory, moreover, when* $\hat{\tau_{c1}}<\tau_{c}<\hat{\tau_{c2}}$*, the optimal decision is R^MV^, otherwise, i.e.,* $\tau_{c}>\hat{\tau_{c2}}$, the optimal decision is R^MV*^, where $\hat{\tau_{c1}}=\frac{\gamma_{2}-\gamma_{1}\delta +e\varepsilon \left(\delta -\alpha \right)}{\delta \left[\gamma_{1}-\gamma_{2}-e\varepsilon \left(1-\alpha \right)\right]}$ *and* $\hat{\tau_{c2}}=\frac{\delta \left[\gamma_{1}-\gamma_{2}-2c_{c}-e\varepsilon \left(1-\alpha \right)\right]+\sqrt{\delta^{2}{\left[\gamma_{1}-\gamma_{2}-2c_{c}-e\varepsilon \left(1-\alpha \right)\right]}^{2}-4c_{c}\delta \left[\gamma_{2}+c_{c}-\gamma_{1}\delta -e\left(\alpha -\delta \right)\varepsilon \right]}}{2c_{c}\delta }$.

The interpretation of Corollary 5 is similar to Corollary 2, but the thresholds of the collection target are associated with carbon emissions. Finally, we will study the optimal decisions of the manufacturer under regulation with both collection and remanufacturing targets, i.e., $\tau_{c}>\tau_{r}>0$.

**Proposition** **6.** *Under regulations with both collection and remanufacturing targets,* ∃ $g_{4}$*, where* $g_{3}>g_{4}>g_{0}$*, such that (i) if* $g_{0}<u\leq g_{4}$*, the optimal solution is RR^MM^; (ii) otherwise (i.e.,* $u>g_{4}$*), the optimal strategies are the same as ones under regulation with collection target alone.*

Figure 3c illustrates the optimal strategies under take-back regulations with both collection and remanufacturing targets. Compared with Figure 3b, the only difference in Figure 3c is that when $g_{0}<u\leq g_{4}$, the optimal strategy of the manufacturer changes from RR^MV*^ to RR^MM^. Proposition 6 shows that under take-back regulations with additional remanufacturing targets, when $u>g_{4}$, the optimal decision is the same as that under regulation only with collection targets, while when $g_{0}<u\leq g_{4}$, the remanufacturing target is also mandatory, i.e., $q_{r}^{*}=\tau_{r}q_{n}^{*}$. Proposition 6 implies that the regulation with additional remanufacturing targets will play a role only when *u* is low and *α* is high.

**Corollary** **6.** ∃ $\hat{\tau_{r}}$*, when* $\tau_{r}>\hat{\tau_{r}}$*, the remanufacturing target is mandatory, and the optimal decision is RR^MM^. Otherwise, additional remanufacturing target has no effect on optimal decisions, and the manufacturer remanufactures voluntarily, where* $\hat{\tau_{r}}=\frac{\gamma_{2}-\gamma_{1}\delta -e\varepsilon \left(\alpha -\delta \right)+c_{c}\left(1+\delta \tau_{c}\right)}{\delta \left(\gamma_{1}-\gamma_{2}-c_{c}-e\varepsilon +e\alpha \varepsilon -c_{c}\tau_{c}\right)}$.

The optimal decisions of the manufacturer are shown in Table 6, and the optimal profits and carbon emissions are shown in Table 7.

## 5. Environmental Impact Analysis

In this section, we will explore the environmental impact of take-back regulations and cap-and-trade regulations. There are many ways to measure the environmental impact, including the use of non-renewable resources, energy consumption or environmental damage during the entire life cycle [44,45], and the release of harmful gases during production, especially the emissions of greenhouse gases [30]. The total amount of greenhouse gas emissions is also an indicator commonly used in industry for environmental assessment [50], hence, we use carbon emissions (also called carbon footprint) [51] to measure the environmental impact. Similar to Yenipazarli [30], we believe that the environmental impact is the carbon emissions of new and remanufactured products during production.

### 5.1. The Impact of Take-Back Regulation

Take-back regulations around the world are constantly changing and requirements are becoming more stringent. For example, the EU WEEE Directive will raise the collection target from 45 to 65% by 2019 [7]. The “Extended Producer Responsibility System Implementation Plan” promulgated by China points out that the collection rate will reach 40% by 2020, and it will reach 50% by 2025 [8]. How will these stricter regulations affect the environment?

Take-back regulations are becoming constantly stricter and the goal is to reduce the environmental impact. Therefore, the government hopes to reduce the environmental impact by raising the collection and remanufacturing targets. However, the following study found that this is not always the case.

As mentioned above, take-back regulations have two targets: a collection target, $\tau_{c}$, and a remanufacturing target, $\tau_{r}$. We will investigate the environmental impact of regulation when imposing higher collection and remanufacturing targets. In order to fully understand the implications of higher targets on the environment, we firstly explore how regulations affect the quantities of new and remanufactured products.

**Lemma** **1.** *(i)* $\frac{\partial q_{n}{}^{*}}{\partial \tau_{c}}\leq 0$*,* $\frac{\partial q_{r}{}^{*}}{\partial \tau_{c}}\geq 0$*. (ii)* $\frac{\partial q_{n}{}^{*}}{\partial \tau_{r}}\leq 0$*,* $\frac{\partial q_{r}{}^{*}}{\partial \tau_{r}}\geq 0$.

Lemma 1 shows that as regulation imposes higher collection and remanufacturing targets, the quantity of new products always decreases, whereas the quantity of remanufactured products increases. Although remanufacturing is considered to be more environmentally friendly than manufacturing, the overall environmental impact cannot be simply obtained. The following Proposition will further explore how collection target affect the environment.

**Proposition** **7.** *The impact of the collection target on the environment is as follows: If* $q_{r}=\tau_{c}q_{n}$ *and* $\alpha >\bar{\alpha }$*, then* $\frac{\partial E^{*}}{\partial \tau_{c}}>0$*, otherwise,* $\frac{\partial E^{*}}{\partial \tau_{c}}\leq 0$*, where* $\bar{\alpha }=\frac{2\delta \left(1+\tau_{c}\right)q_{n}+\lambda_{4}}{2\left(1+\delta \tau_{c}\right)q_{n}-\lambda_{4}\tau_{c}}$.

Proposition 7 confirms us that if $q_{r}<\tau_{c}q_{n}$, the total environmental impact decreases with the increase of collection target $\tau_{c}$. However, the result may be different when $q_{r}=\tau_{c}q_{n}$. In this case, if emissions intensity of remanufacturing, *α*, is sufficiently high, i.e., $\alpha >\bar{\alpha }$, the increase of collection target may increase total environmental impact. Proposition 7 implies that stricter regulation might actually lead to inferior environmental outcomes.

The purpose of an increased collection target is to reduce the impact on the environment. However, unexpectedly, the study found that when take-back regulation is binding (i.e., $q_{r}=\tau_{c}q_{n}$) and the environmental impact of remanufacturing is high (i.e., $\alpha >\bar{\alpha }$), with the increase of collection target, the total carbon emissions during production increase. In other words, the increase of collection target may even aggravate the environmental impact.

**Proposition** **8.** *The impact of the remanufacturing target on the environment is as follows: If* $q_{r}=\tau_{r}q_{n}$ *and* $\alpha >\bar{\bar{\alpha }}$*, then* $\frac{\partial E^{*}}{\partial \tau_{r}}>0$*, otherwise,* $\frac{\partial E^{*}}{\partial \tau_{r}}\leq 0$*, where* $\bar{\bar{\alpha }}=\frac{2\delta \left(1+\tau_{r}\right)q_{n}+\lambda_{3}}{2\left(1+\delta \tau_{r}\right)q_{n}-\lambda_{3}\tau_{r}}$.

Proposition 8 implies that as remanufacturing targets become stricter, the manufacturer reduces the quantity of new products but increases the quantity of remanufactured products, leading to an increased total amount of products. If the relative environmental impact of remanufacturing is sufficiently high, i.e., $\alpha >\bar{\bar{\alpha }}$, the total environmental impact increases too.

Even though the increase of collection and remanufacturing targets might lead to inferior environmental outcomes under certain circumstances, however, it is good for the environment under the other circumstances, hence the government should not abandon take-back regulation. Instead, policy makers need to be more cautious about potential unintended consequences and may need to consider supporting take-back legislation with other environmental policies such as energy-efficiency requirements and introduction of emission reduction technologies.

### 5.2. The Impact of Cap-and-Trade Regulation

**Proposition** **9.** *(i) Carbon emissions have been reduced by the introduction of carbon cap-and-trade regulation. (ii) The total carbon emissions decrease as the price of carbon trading increases, i.e.,* $\frac{\partial E^{*}}{\partial \varepsilon }<0$.

Proposition 9 indicates it is friendlier to the environment when cap-and-trade regulations are considered. In other words, the carbon emissions decrease due to the implementation of carbon cap-and-trade regulations. On the other hand, with the increase of carbon emissions trading price, the environmental impact decreases, hence government regulation of carbon emissions trading price is an effective way to reduce environmental impact. These conclusions are consistent with the research of [31,34].

## 6. Numerical Analysis

In this chapter, we will use a numerical example to illustrate theoretical results more effectively under both carbon cap-and-trade regulation and take-back regulation. We firstly analyze the impact of collection target, $\tau_{c}$, remanufacturing target, $\tau_{r}$, and cost saving of remanufacturing, u, on the optimal decisions of the manufacturer. Consequently, we explore how environmental impact change with respect $\tau_{c}$, $\tau_{r}$ and α. The parameters of the example are set as follows: $c_{n}=0.4$, $c_{c}=0.05$, u=0.25, δ=0.5, e=0.5, ε=0.2, $\tau_{c}=0.6$, $\tau_{r}=0.2$ and α=0.5.

**Figure 4:**
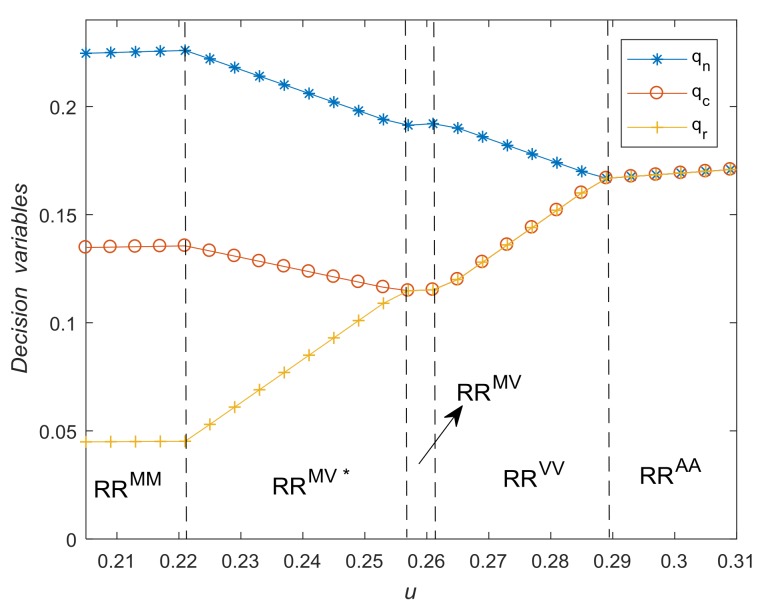
The impact of *u* on optimal decisions.

The cost saving of remanufacturing, u, plays a key role in the choosing of optimal strategies. Figure 4 shows how should the manufacturer make optimal decisions according to u. When $\tau_{c}=0.5$ and $\tau_{r}=0.2$, thresholds of u are $g_{0}=0.2050$, $g_{4}=0.2211$, $g_{3}=0.2558$, $g_{2}=0.2627$, $g_{1}=0.2883$. Figure 4 also illustrates the impact of u on $q_{n}^{*}$, $q_{c}^{*}$ and $q_{r}^{*}$. With the increase of u, the quantity of remanufactured products increases whereas the quantity of new products decreases. More specifically, when $u>g_{1}$, the optimal solution is *RR^AA^*, i.e., $q_{n}^{*}=q_{c}^{*}=q_{r}^{*}$. When $g_{2}<u<g_{1}$, the optimal solution is *RR^VV^*, i.e., $\frac{q_{c}}{q_{n}}>\tau_{c}=0.5$, which means the collection target is redundant. When $g_{3}<u<g_{2}$, the optimal solution is *RR^MV^*, i.e., $\frac{q_{c}}{q_{n}}=\tau_{c}=0.5$, in this case, all the collected WEEE is used for remanufacturing. While in solution RR^MV*^, only part of collected WEEE is used for remanufacturing. Finally, when $g_{0}<u<g_{4}$, the optimal solution is *RR^M.M^* i.e., $\frac{q_{r}}{q_{n}}=\tau_{r}=0.2$. These findings verify Proposition 5 and Proposition 6.

**Figure 5:**
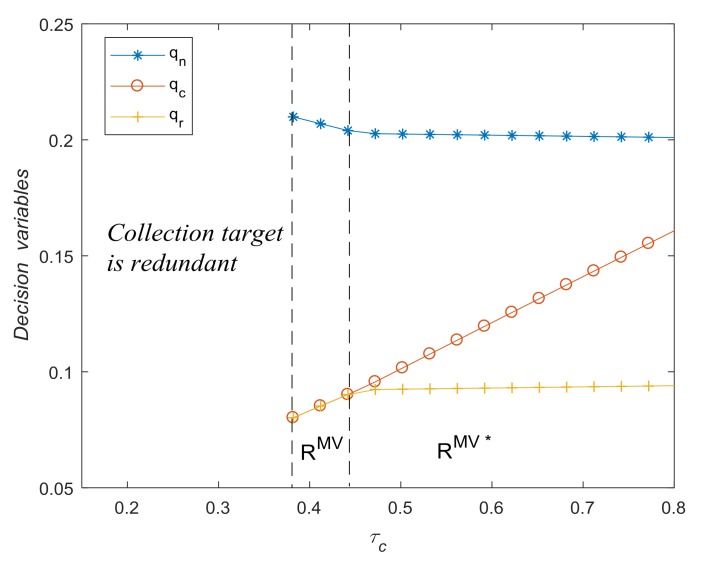
The impact of $\tau_{c}$ on optimal decisions.

Figure 5 shows the impact of $\tau_{c}$ on optimal decisions $q_{n}^{*}$, $q_{c}^{*}$ and $q_{r}^{*}$. The tresholds of the collection target $\tau_{c}$ are $\hat{\tau_{c1}}=0.3810$ and $\hat{\tau_{c2}}=0.4608$. When $\tau_{c}<\hat{\tau_{c1}}$, take-back regulations with a collection target have no effect on optimal decisions, and the manufacturer collects WEEE voluntarily, which means the collection target is redundant. When $\hat{\tau_{c1}}<\tau_{c}<\hat{\tau_{c2}}$, the optimal solution is *R^MV^*, i.e., $q_{c}^{*}=q_{r}^{*}=\tau_{c}q_{n}^{*}$. When $\tau_{c}>\hat{\tau_{c2}}$, the optimal solution is R^MV*^, i.e., $q_{r}^{*}<q_{c}^{*}=\tau_{c}q_{n}^{*}$. Figure 5 also shows that with the increase of $\tau_{c}$, both the quantity of remanufactured products and collection amount increase whereas the quantity of new products decreases. These findings verify Corollary 5 and Lemma 1 (i).

**Figure 6:**
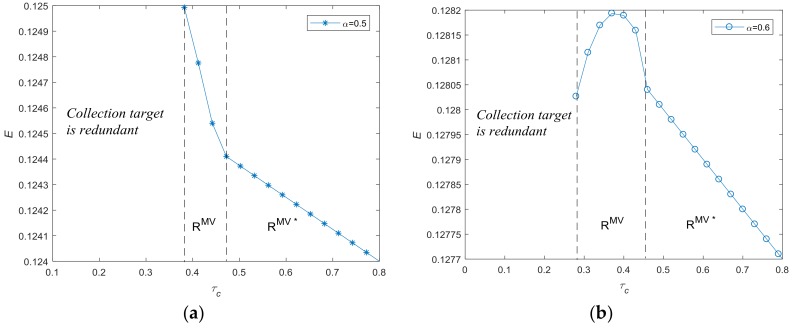
Environmental impact with respect to $\tau_{c}$ and α. (**a**) when α=0.5; (**b**) when α=0.6.

Figure 6 shows how environmental impact change with respect to $\tau_{c}$ and α. When α=0.6, thresholds of collection target $\tau_{c}$ are $\hat{\tau_{c1}}=0.2727$ and $\hat{\tau_{c2}}=0.4422$. In both cases, when $q_{r}<\tau_{c}q_{n}$ (i.e., solution R^MV*^), environmental impact decreases with the increase of collection target $\tau_{c}$. When α=0.5 and $q_{r}=\tau_{c}q_{n}$ (i.e., solution R^MV*^ in Figure 6a), environmental impact decreases with the increase of $\tau_{c}$ as well. However, When α=0.6 and $q_{r}=\tau_{c}q_{n}$ (i.e., solution R^MV*^ in Figure 6b), the environmental impact may even increase with the increase of $\tau_{c}$. This finding verifies Proposition 7.

**Figure 7:**
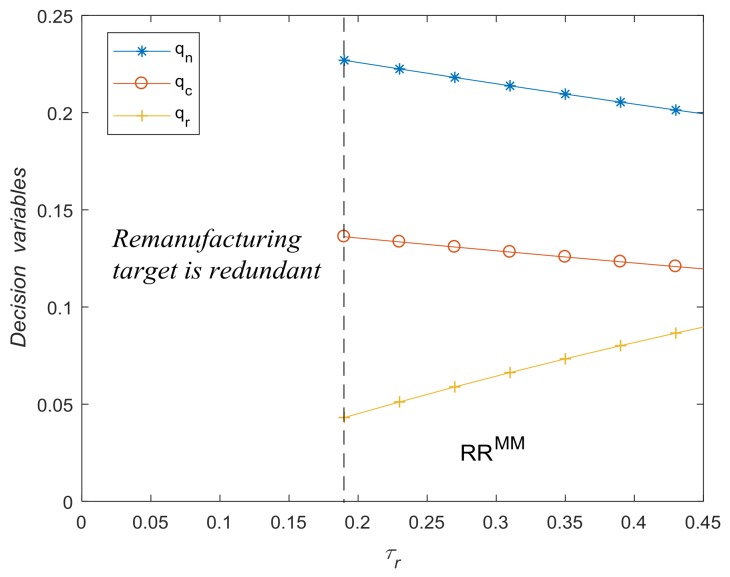
The impact of $\tau_{r}$ on optimal decisions.

Figure 7 illustrates the impact of $\tau_{r}$ on $q_{n}^{*}$, $q_{c}^{*}$ and $q_{r}^{*}$. The threshold of remanufacturing target is $\hat{\tau_{r}}=0.1894$. When $\tau_{r}<\hat{\tau_{r}}$, take-back regulation with additional remanufacturing target has no effect on optimal decision, and manufacturer enters remanufacturing market voluntarily, which means the remanufacturing target is redundant. When $\tau_{c}>\hat{\tau_{r}}$, the optimal solution is *RR^MM^*, i.e., $q_{c}^{*}=\tau_{c}q_{n}^{*}$ and $\;q_{r}^{*}=\tau_{r}q_{n}^{*}$. Figure 7 also shows that with the increase of $\tau_{r}$, the quantity of remanufactured products increases whereas the quantity of new products and collection amount decrease. These findings verify Corollary 6 and Lemma 1 (ii).

**Figure 8:**
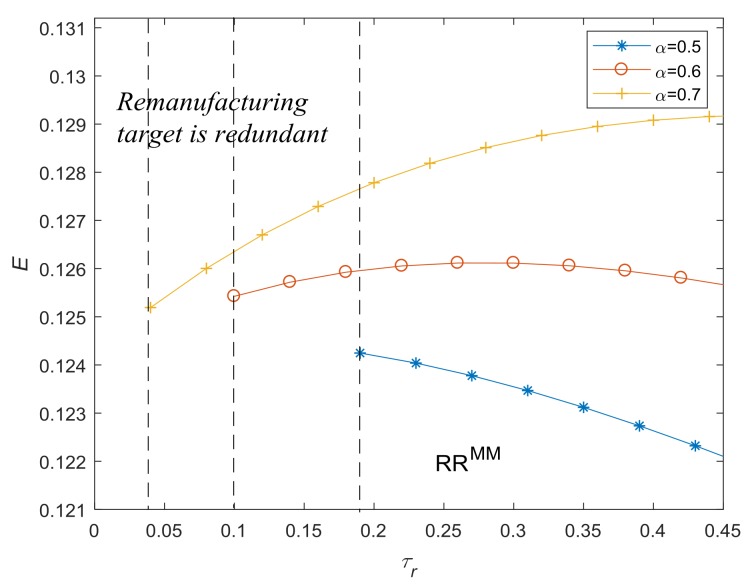
Environmental impact with respect to $\tau_{r}$ and α.

Figure 8 shows how the environmental impact change with respect to $\tau_{r}$ and α. We set α as 0.5, 0.6 and 0.7, respectively, and thresholds of remanufacturing target, $\tau_{r}$, are correspondingly 0.1894, 0.0970 and 0.0121. When α=0.5 and $q_{r}=\tau_{r}q_{n}$ (i.e., solution R^MV*^), the environmental impact decreases with the increase of $\tau_{r}$. However, when α=0.7 and $q_{r}=\tau_{r}q_{n}$, the environmental impact even increases with the increase of $\tau_{r}$. This finding verifies Proposition 8.

## 7. Conclusions

As the rapid growth of WEEE is attracting more attention, laws to guide the collection and remanufacturing of the manufacturer are particularly urgent. In order to reduce the impact of WEEE landfilling on the environment and realize the effective reuse of resources, government have promulgated take-back regulations. On the other hand, global warming has attracted more attention in reducing carbon emissions. In order to reduce carbon emissions during the production phase, governments have enacted cap-and-trade regulations. 

This paper incorporates take-back regulations and cap-and-trade regulations into the production and collection decisions of a monopoly manufacturer who produces both new and remanufactured products. We firstly analyse the optimal decisions of the manufacturer under different regulatory scenarios (with/without cap-and-trade regulation + with/without take-back regulation), and then explore the environmental impact with the implementation of carbon cap-and-trade regulation and more stringent take-back regulation. 

By comparing all the analysis above, we obtain some interesting managerial insights:

(1) Under carbon cap-and-trade regulations and take-back regulations with both collection and remanufacturing targets, there are five optimal solutions for the manufacturer: solution *RR^AA^* (i.e., $q_{n}^{*}=q_{c}^{*}=q_{r}^{*}$), solution *RR^VV^* (i.e., $q_{n}^{*}>q_{r}^{*}=q_{c}^{*}>\tau_{c}q_{n}^{*}$), solution *RR^MV^* (i.e., $\tau_{c}q_{n}^{*}=q_{c}^{*}=q_{r}^{*}>\tau_{r}q_{n}^{*}$), solution RR^MV*^ (i.e., $\tau_{c}q_{n}^{*}=q_{c}^{*}>q_{r}^{*}>\tau_{r}q_{n}^{*}$), and solution *RR^AA^* (i.e., $\tau_{c}q_{n}^{*}=q_{c}^{*}>q_{r}^{*}=\tau_{r}q_{n}^{*}$). Furthermore, bounds describing the characteristics of the optimal solutions are obtained;
(2) In the absence of carbon cap-and-trade regulations, when both the cost savings of remanufacturing, *u*, and consumer value discount for the remanufactured products, *δ*, are quite high, the manufacturer collects WEEE voluntarily i.e., the collection target is redundant. Furthermore, the regulation with additional remanufacturing target will play a role only when *u* and *δ* are relatively low;
(3) Under carbon cap-and-trade regulations, when the cost saving of a remanufactured product, *u*, is high and emissions intensity of a remanufactured (vis-á-vis new) product, *α*, is low, the manufacturer collects WEEE voluntarily i.e., the collection target is redundant. Furthermore, the regulations with additional remanufacturing targets will play a role only when *u* is low and *α* is high;
(4) It is friendlier to the environment when cap-and-trade regulations are considered, in other words, carbon cap-and-trade regulations can reduce carbon emissions. What’s more, with the increase of carbon trading price, the environmental impact decreases, therefore, for the government, thsis is an effective way to reduce environmental impact by regulating the price of carbon trading;
(5) As collection and remanufacturing targets of take-back regulations become stricter, the manufacturer reduces the production of new products and increases the quantity of remanufactured products, leading to an increase of total production. Unexpectedly, the total environmental impact might even increase, if the emission intensity of a remanufactured (vis-á-vis a new product) is sufficiently high.

Even though the increase of collection and remanufacturing targets might lead to inferior environmental outcomes (i.e., more carbon emissions), our results do not mean that government should abandon take-back regulation. Instead, policy makers simply need to be more cautious about potential unintended consequences and may need to consider supporting take-back legislation with other environmental policies such as energy-efficiency requirements and the introduction of emission reduction technologies.

This paper has some limitations. First, we study the decisions of a monopoly manufacturer, scilicet, there is a complete lack of market competition and the manufacturer can act independently. However, as remanufacturing WEEE has a net profit, the remanufacturer will enter the remanufacturing market. Naturally, external competition should be considered, such as competition between remanufacturers and manufacturers. Second, this paper is based on the assumption of complete information and demand determination. If asymmetric information or market demand is taken into account, the problem will be more complicated. Finally, we consider individual take-back schemes, but most take-back regulations are of the collective variety. In order to match the real-world situation, it is necessary to extend our study to the collective situation.

## Figures and Tables

**Table 1:** The major parameters and notations.

Notation	Definition
Parameters	
$p_{n}$,$p_{r}$	Sale prices of new products and remanufactured products
$c_{n}$,$c_{r}$,$c_{c}$	Unit cost of manufacturing, remanufacturing and collection
$u=c_{n}-c_{r}$	Cost saving of unit remanufactured product
$\tau_{c}$	Collection target prescribed by the government
$\tau_{r}$	Remanufacturing target prescribed by the government
e	Carbon emissions of unit new product
α	Emission intensity of a remanufactured (vis-á-vis new) product
E	Total carbon emissions
θ	Consumers’ willingness to pay for new products
δ	Value discount of consumer for the remanufactured products
ε	Unit carbon trading price
K	Carbon emission cap
Decision variables	
$q_{n}$,$q_{r}$,$q_{c}$	Manufacturing quantity, remanufacturing quantity, collection mount
Objective function	
$\Pi^{X}$	Total profit of the manufacturer, X∈{NR,R,RR}

**Table 2:** Solutions and explanation.

Solution	Notation (Superscript)	Explanation
1	*MM*	$\tau_{c}q_{n}^{*}=q_{c}^{*}>q_{r}^{*}=\tau_{r}q_{n}^{*}$
2	MV*	$\tau_{c}q_{n}^{*}=q_{c}^{*}>q_{r}^{*}>\tau_{r}q_{n}^{*}$
3	*MV*	$\tau_{c}q_{n}^{*}=q_{c}^{*}=q_{r}^{*}>\tau_{r}q_{n}^{*}$
4	*VV*	$q_{n}^{*}>q_{r}^{*}=q_{c}^{*}>\tau_{c}q_{n}^{*}$
5	*AA*	$q_{n}^{*}=q_{c}^{*}=q_{r}^{*}$

**Table 3:** Bounds of *u* describing the characteristics of the optimal solutions.

Bounds	Expression	Bounds	Expression
$t_{0}$	$\;c_{c}+c_{n}-c_{n}\delta$	$g_{0}$	$\;c_{c}+c_{n}-c_{n}\delta +e\left(\alpha -\delta \right)\varepsilon$
$t_{1}$	$\frac{c_{c}\left(1+\delta \right)+\left(1-\delta \right)\left(c_{n}+\delta \right)}{1+\delta }$	$g_{1}$	$\alpha e\varepsilon +\frac{c_{c}\left(1+\delta \right)+\left(1-\delta \right)\left(c_{n}+\delta \right)-2e\delta \varepsilon }{1+\delta }$
$t_{2}$	$1+c_{c}-\delta -\frac{\left(1-\delta \right)\left(1-c_{n}\right)}{1+\delta \tau_{c}}$	$g_{2}$	$e\alpha \varepsilon +1+c_{c}-\delta -e\varepsilon -\frac{\left(1-\delta \right)\left(1-c_{n}-e\varepsilon \right)}{1+\delta \tau_{c}}$
$t_{3}$	$\frac{c_{n}-c_{n}\delta +\delta \tau_{c}\left(1-c_{c}-\delta -c_{c}\tau_{c}\right)}{1+\delta \tau_{c}}$	$g_{3}$	$\alpha e\varepsilon +\frac{c_{n}-c_{n}\delta -e\delta \varepsilon -e\delta \varepsilon \tau_{c}+\delta \tau_{c}\left(1-c_{c}-\delta -c_{c}\tau_{c}\right)}{1+\delta \tau_{c}}$
$t_{4}$	$\frac{c_{n}-c_{n}\delta +\left(1-\delta \right)\delta \tau_{r}-c_{c}\delta \tau_{c}\left(1+\tau_{r}\right)}{1+\delta \tau_{r}}$	$g_{4}$	$\alpha e\varepsilon +\frac{c_{n}-c_{n}\delta -e\delta \varepsilon +\left(1-\delta \right)\delta \tau_{r}-e\delta \varepsilon \tau_{r}-c_{c}\delta \tau_{c}\left(1+\tau_{r}\right)}{1+\delta \tau_{r}}$

**Table 4:** Optimal decisions of the manufacturer in absence of cap-and-trade regulation.

Solutions	$q_{n}{}^{*}$	$q_{c}{}^{*}$	$q_{r}{}^{*}$
Solution 1 (*R^MM^*)	$\frac{\gamma_{1}-c_{c}\tau_{c}+\left(c_{c}+\gamma_{2}\right)\tau_{r}}{2+2\delta \tau_{r}\left(2+\tau_{r}\right)}$	$\tau_{c}q_{n}{}^{*}$	$\tau_{r}q_{n}{}^{*}$
Solution 2 (R^MV*^, RR^MV*^)	$\frac{\gamma_{1}-\gamma_{2}-c_{c}-c_{c}\tau_{c}}{2\left(1-\delta \right)}$	$\tau_{c}q_{n}{}^{*}$	$\frac{\gamma_{2}+c_{c}}{2\delta }-q_{n}{}^{*}$
Solution 3 (*R^MV^*, *RR^MV^*)	$\frac{\gamma_{1}+\gamma_{2}\tau_{c}}{2+2\delta \tau_{c}\left(2+\tau_{c}\right)}$	$\tau_{c}q_{n}{}^{*}$	$q_{c}{}^{*}$
Solution 4 (*NR^VV^*, *R^VV^*, *RR^VV^*)	$\frac{\gamma_{1}-\gamma_{2}}{2\left(1-\delta \right)}$	$\frac{\gamma_{2}}{2\delta }-q_{n}{}^{*}$	$q_{c}{}^{*}$
Solution 5 (*NR^AA^*, *R^AA^*, *RR^AA^*)	$\frac{\gamma_{1}+\gamma_{2}}{2+6\delta }$	$q_{n}{}^{*}$	$q_{n}{}^{*}$

**Table 5:** Optimal profits and carbon emissions in absence of cap-and-trade regulation.

Solutions	$E^{*}$	$\Pi^{*}$
Solution 1 (*R^MM^*)	$\frac{e\left(1+\alpha \tau_{r}\right)\left[\gamma_{1}-c_{c}\tau_{c}+\left(c_{c}+\gamma_{2}\right)\tau_{r}\right]}{2+2\delta \tau_{r}\left(2+\tau_{r}\right)}$	$\frac{{\left[\gamma_{1}-c_{c}\tau_{c}+\left(c_{c}+\gamma_{2}\right)\tau_{r}\right]}^{2}}{4+4\delta \tau_{r}\left(2+\tau_{r}\right)}$
Solution 2 (R^MV*^, RR^MV*^)	$\frac{e\left(1-\alpha \right)\left[\gamma_{1}-\gamma_{2}-c_{c}-c_{c}\tau_{c}\right]}{2\left(1-\delta \right)}+\frac{e\alpha \left(\gamma_{2}+c_{c}\right)}{2\delta }$	$\frac{{\left[\gamma_{1}-\gamma_{2}-c_{c}-c_{c}\tau_{c}\right]}^{2}}{4\left(1-\delta \right)}+\frac{{\left(\gamma_{2}+c_{c}\right)}^{2}}{4\delta }$
Solution 3 (*R^MV^*, *RR^MV^*)	$\frac{e\left(1+\alpha \tau_{c}\right)\left(\gamma_{1}+\gamma_{2}\tau_{c}\right)}{2+2\delta \tau_{c}\left(2+\tau_{c}\right)}$	$\frac{{\left(\gamma_{1}+\gamma_{2}\tau_{c}\right)}^{2}}{4+4\delta \tau_{c}\left(2+\tau_{c}\right)}$
Solution 4 (*NR^VV^*, *R^VV^*, *RR^VV^*)	$\frac{e\left(1-\alpha \right)\left(\gamma_{1}-\gamma_{2}\right)}{2\left(1-\delta \right)}$	$\frac{\gamma_{2}{}^{2}}{4\delta }+\frac{{\left(\gamma_{1}-\gamma_{2}\right)}^{2}}{4\left(1-\delta \right)}$
Solution 5 (*NR^AA^*, *R^AA^*, *RR^AA^*)	$\frac{e\left(1+\alpha \right)\left(\gamma_{1}+\gamma_{2}\right)}{2+6\delta }$	$\frac{{\left(\gamma_{1}+\gamma_{2}\right)}^{2}}{4+12\delta }$

**Table 6:** Optimal decisions of the manufacturer considering carbon cap-and-trade regulation.

Solutions	$q_{n}{}^{*}$	$q_{c}{}^{*}$	$q_{r}{}^{*}$
Solution 1 (*R^MM^*)	$\frac{\gamma_{1}-c_{c}\tau_{c}+\left(c_{c}+\gamma_{2}\right)\tau_{r}-e\varepsilon \left(1+\alpha \tau_{r}\right)}{2+2\delta \tau_{r}\left(2+\tau_{r}\right)}$	$\tau_{c}q_{n}{}^{*}$	$\tau_{r}q_{n}{}^{*}$
Solution 2 (R^MV*^, RR^MV*^)	$\frac{\gamma_{1}-\gamma_{2}-c_{c}-c_{c}\tau_{c}-e\varepsilon \left(1-\alpha \right)}{2\left(1-\delta \right)}$	$\tau_{c}q_{n}{}^{*}$	$\frac{\gamma_{2}+c_{c}-e\alpha \varepsilon }{2\delta }-q_{n}{}^{*}$
Solution 3 (*R^MV^*, *RR^MV^*)	$\frac{\gamma_{1}+\gamma_{2}\tau_{c}-e\varepsilon \left(1+\alpha \tau_{c}\right)}{2+2\delta \tau_{c}\left(2+\tau_{c}\right)}$	$\tau_{c}q_{n}{}^{*}$	$q_{c}{}^{*}$
Solution 4 (*NR^VV^*, *R^VV^*, *RR^VV^*)	$\frac{\gamma_{1}-\gamma_{2}-e\varepsilon \left(1-\alpha \right)}{2\left(1-\delta \right)}$	$\frac{\gamma_{2}-e\alpha \varepsilon }{2\delta }-q_{n}{}^{*}$	$q_{c}{}^{*}$
Solution 5 (*NR^AA^*, *R^AA^*, *RR^AA^*)	$\frac{\gamma_{1}+\gamma_{2}-e\left(1+\alpha \right)\varepsilon }{2+6\delta }$	$q_{n}{}^{*}$	$q_{n}{}^{*}$

**Table 7:** Optimal profits and carbon emissions considering carbon cap-and-trade regulation.

Solutions	$E^{*}$	$\Pi^{*}$
Solution 1 (*R^MM^*)	$\frac{e\left(1+\alpha \tau_{r}\right)\left[\gamma_{1}-c_{c}\tau_{c}+\left(c_{c}+\gamma_{2}\right)\tau_{r}-e\varepsilon \left(1+\alpha \tau_{r}\right)\right]}{2+2\delta \tau_{r}\left(2+\tau_{r}\right)}$	$\frac{{\left[\gamma_{1}-c_{c}\tau_{c}+\left(c_{c}+\gamma_{2}\right)\tau_{r}-e\varepsilon \left(1+\alpha \tau_{r}\right)\right]}^{2}}{4+4\delta \tau_{r}\left(2+\tau_{r}\right)}+\varepsilon K$
Solution 2 (R^MV*^, RR^MV*^)	$\begin{array}{l} \frac{e\left(1-\alpha \right)\left[\gamma_{1}-\gamma_{2}-c_{c}-c_{c}\tau_{c}-e\varepsilon \left(1-\alpha \right)\right]}{2\left(1-\delta \right)}\\ +\frac{e\alpha \left(\gamma_{2}+c_{c}-e\alpha \varepsilon \right)}{2\delta } \end{array}$	$\begin{array}{l} \frac{{\left[\gamma_{1}-\gamma_{2}-c_{c}-c_{c}\tau_{c}-e\varepsilon \left(1-\alpha \right)\right]}^{2}}{4\left(1-\delta \right)}\\ +\frac{{\left(\gamma_{2}+c_{c}-e\alpha \varepsilon \right)}^{2}}{4\delta }+\varepsilon K \end{array}$
Solution 3 (*R^MV^*, *RR^MV^*)	$\frac{e\left(1+\alpha \tau_{c}\right)\left[\gamma_{1}+\gamma_{2}\tau_{c}-e\varepsilon \left(1+\alpha \tau_{c}\right)\right]}{2+2\delta \tau_{c}\left(2+\tau_{c}\right)}$	$\frac{{\left[\gamma_{1}+\gamma_{2}\tau_{c}-e\varepsilon \left(1+\alpha \tau_{c}\right)\right]}^{2}}{4+4\delta \tau_{c}\left(2+\tau_{c}\right)}+\varepsilon K$
Solution 4 (*NR^VV^*, *R^VV^*, *RR^VV^*)	$\frac{e\alpha \left(\gamma_{2}-\alpha e\varepsilon \right)}{2\delta }+\frac{e\left(1-\alpha \right)\left[\gamma_{1}-\gamma_{2}-e\varepsilon \left(1-\alpha \right)\right]}{2\left(1-\delta \right)}$	$\frac{{\left(\gamma_{2}-\alpha e\varepsilon \right)}^{2}}{4\delta }+\frac{{\left[\gamma_{1}-\gamma_{2}-e\varepsilon \left(1-\alpha \right)\right]}^{2}}{4\left(1-\delta \right)}+\varepsilon K$
Solution 5 (*NR^AA^*, *R^AA^*, *RR^AA^*)	$\frac{e\left(1+\alpha \right)\left[\gamma_{1}+\gamma_{2}-e\varepsilon \left(1+\alpha \right)\right]}{2+6\delta }$	$\frac{{\left[\gamma_{1}+\gamma_{2}-e\varepsilon \left(1+\alpha \right)\right]}^{2}}{4+12\delta }+\varepsilon K$

## Appendix A. Proof of propositions and Corollaries

**Proof of Proposition 1.** The Lagrange function is $L_{}^{NR}=\Pi^{NR}+\lambda_{1}\left(q_{n}-q_{c}\right)+\lambda_{2}\left(q_{c}-q_{r}\right)+\lambda_{3}q_{r}$, where $\lambda_{i}\geq 0$, i=1,2,3 are Lagrange multipliers. The corresponding Hessian Matrix is $H=\left[\begin{array}{ccc} -2 & -2\delta & 0\\ -2\delta & -2\delta & 0\\ 0 & 0 & 0 \end{array}\right]$, as $\Delta_{1}=-2<0$, $\Delta_{2}=4\delta (1-\delta )>0$, and |H|=0 the Hessian Matrix is negative semidefinite, and thus the first-order conditions guarantee optimality. Then, solving the Karush-Kuhn-Tucker (KKT) conditions, $\frac{\partial L_{}^{NR}}{\partial q_{n}}=\gamma_{1}-2q_{n}-2q_{r}\delta t+\lambda_{1}=0$, $\frac{\partial L_{}^{NR}}{\partial q_{r}}=\gamma_{2}+c_{c}-2q_{n}\delta -2q_{r}\delta t-\lambda_{2}+\lambda_{3}=0$, $\frac{\partial L_{}^{NR}}{\partial q_{c}}=-c_{c}-\lambda_{1}+\lambda_{2}=0$, $\lambda_{1}\left(q_{n}-q_{c}\right)=0$, $\lambda_{2}\left(q_{c}-q_{r}\right)=0$, $\lambda_{3}q_{r}=0$, $\lambda_{i}\geq 0,\;i=1,2,3$, $q_{n}-q_{c}\geq 0$, $q_{c}-q_{r}\geq 0$ and $q_{r}\geq 0$, we can get three solutions:

Solution 1: Here, $q_{n}^{*}=\frac{1}{2}\gamma_{1}$, $q_{c}^{*}=q_{r}^{*}=0$, $\lambda_{1}^{*}=0$, $\lambda_{2}^{*}=c_{c}$ and $\lambda_{3}^{*}=\gamma_{1}\delta -\gamma_{2}$, the manufacturer only produces new products, however, we only focused on the situation that new and remanufactured products coexist.

Solution 2 (*NR^VV^*): Here, $q_{n}^{*}>q_{c}^{*}=q_{r}^{*}\text{>}$, $\lambda_{1}^{*}=\lambda_{3}^{*}=0$ and $\lambda_{2}^{*}=c_{c}$. The optimality condition $q_{r}^{*}>0$ implies $u>t_{0}$, while $q_{r}^{*}<q_{n}^{*}$ implies $u<t_{1}$.

Solution 3 (*NR^AA^*): Here, $q_{n}^{*}=q_{c}^{*}=q_{r}^{*}\text{>}$, $\lambda_{1}^{*}=\frac{\gamma_{2}-2\gamma_{1}\delta +\gamma_{2}\delta }{1+3\delta }$, $\lambda_{2}^{*}=c_{c}-\gamma_{1}+\frac{\left(1+\delta \right)\left(\gamma_{1}+\gamma_{2}\right)}{1+3\delta }$ and $\lambda_{3}^{*}=0$. The optimality condition $\lambda_{1}^{*}\geq 0$ can be written as $u\geq t_{1}$.□

**Proof of Corollary 1.** According to $u>t_{0}$, we get $\frac{\gamma_{2}}{\gamma_{1}}>\delta$. □

**Proof of Proposition 2.** The Lagrange function is $L_{}^{R}=\Pi^{R}+\lambda_{1}\left(q_{n}-q_{c}\right)+\lambda_{2}\left(q_{c}-q_{r}\right)+\lambda_{3}q_{r}+\lambda_{4}\left(q_{c}-\tau_{c}q_{n}\right)$. The Hessian is negative semidefinite, and thus the first-order conditions guarantee optimality. Then, solving the Karush-Kuhn-Tucker (KKT) conditions, $\frac{\partial L_{}^{R}}{\partial q_{n}}=\gamma_{1}-2q_{n}-2q_{r}\delta +\lambda_{1}-\lambda_{4}\tau_{c}=0$, $\frac{\partial L_{}^{R}}{\partial q_{r}}=\gamma_{2}+c_{c}-2q_{n}\delta -2q_{r}\delta -\lambda_{2}+\lambda_{3}=0$, $\frac{\partial L_{}^{R}}{\partial q_{c}}=-c_{c}-\lambda_{1}+\lambda_{2}+\lambda_{4}=0$, $\lambda_{1}\left(q_{n}-q_{c}\right)=0$, $\lambda_{2}\left(q_{c}-q_{r}\right)=0$, $\lambda_{3}q_{r}=0$, $\lambda_{4}\left(q_{c}-\tau_{c}q_{n}\right)=0$, $\lambda_{i}\geq 0,\;i=1,2,3,4$, $q_{n}>q_{c}$, $q_{c}>q_{r}$, $q_{r}>0$ and $q_{c}>\tau_{c}q_{n}$, after eliminate the redundant solutions, we get five solutions:

Solution 1: $q_{r}^{*}=0$, we don’t consider this case, as we assume the manufacturer participates in remanufacturing in the absence of regulation.

Solution 2 (*R^MV*^*): Here, $q_{c}^{*}=\tau_{c}q_{n}^{*}>q_{r}^{*}\text{>}$, $\lambda_{1}^{*}=\lambda_{2}^{*}=\lambda_{3}^{*}=0$ and $\lambda_{4}^{*}=c_{c}$. The optimality condition $q_{c}^{*}>q_{r}^{*}$ implies $u<t_{3}$.

Solution 3 (*R^MV^*): Here, $q_{r}^{*}=q_{c}^{*}=\tau_{c}q_{n}^{*}$, $\lambda_{1}^{*}=\lambda_{3}^{*}=0$, $\lambda_{2}^{*}=\gamma_{2}+c_{c}-\frac{\delta \left(1+\tau_{c}\right)\left(\gamma_{1}+\gamma_{2}\tau_{c}\right)}{1+\delta \tau_{c}\left(2+\tau_{c}\right)}$, and $\lambda_{4}^{*}=\frac{\gamma_{1}\delta \left(1+\tau_{c}\right)-\gamma_{2}\left(1+\delta \tau_{c}\right)}{1+\delta \tau_{c}\left(2+\tau_{c}\right)}$. The optimality condition $\lambda_{2}^{*}\geq 0$ can be written as $u\geq t_{3}$, while $\lambda_{4}^{*}\geq 0$ can be written as $u\leq t_{2}$ ($t_{2}>t_{3}$).

Solution 4 (*R^VV^*): Here, $q_{n}^{*}>q_{r}^{*}=q_{c}^{*}\text{>}\tau_{c}q_{n}^{*}$, $\lambda_{1}^{*}=\lambda_{3}^{*}=\lambda_{4}^{*}=0$, and $\lambda_{2}^{*}=c_{c}$. The optimality conditions $q_{c}^{*}\text{>}\tau_{c}q_{n}^{*}$ implies $u>g_{2}$, while $q_{r}^{*}<q_{n}^{*}$ implies $u<g_{1}$, furthermore $t_{1}>t_{2}$ (as $\tau_{c}<1$). Note that this is the same as solution 2 under no regulation (*NR^VV^*).

Solution 5 (*R^AA^*): Here, $q_{r}^{*}=q_{c}^{*}=q_{n}^{*}$, $\lambda_{1}^{*}=\frac{\gamma_{2}-2\gamma_{1}\delta +\gamma_{2}\delta }{1+3\delta }$, $\lambda_{2}^{*}=c_{c}-\gamma_{1}+\frac{\left(1+\delta \right)\left(\gamma_{1}+\gamma_{2}\right)}{1+3\delta }$ and $\lambda_{3}^{*}=\lambda_{4}^{*}=0$. The optimality condition $\lambda_{1}^{*}\geq 0$ can be written as $u\geq t_{1}$. Note that this is the same as solution 3 under no regulation (i.e., *NR^AA^*). □

**Proof of Corollary 2.** According to $u=1+c_{c}-\delta -\frac{\left(1-\delta \right)\left(1-c_{n}\right)}{1+\delta \tau_{c}}$, we have $\tau_{c1}$. And according to $u=\frac{c_{n}-c_{n}\delta +\delta \tau_{c}\left(1-c_{c}-\delta -c_{c}\tau_{c}\right)}{1+\delta \tau_{c}}$, we have $\tau_{c2}$. □

**Proof of Proposition 3.** The Lagrange function is $L_{}^{RR}=\Pi^{RR}+\lambda_{1}\left(q_{n}-q_{c}\right)+\lambda_{2}\left(q_{c}-q_{r}\right)+\lambda_{3}\left(q_{r}-\tau_{r}q_{n}\right)+\lambda_{4}\left(q_{c}-\tau_{c}q_{n}\right)$. The Hessian is negative semidefinite, and thus the first-order conditions guarantee optimality. Then, solving the Karush-Kuhn-Tucker (KKT) conditions, $\frac{\partial L_{}^{RR}}{\partial q_{n}}=\gamma_{1}-2q_{n}-2q_{r}\delta +\lambda_{1}-\lambda_{3}\tau_{r}-\lambda_{4}\tau_{c}=0$, $\frac{\partial L_{}^{RR}}{\partial q_{r}}=\gamma_{2}+c_{c}-2\delta q_{n}-2\delta q_{r}-\lambda_{2}+\lambda_{3}=0$, $\frac{\partial L_{}^{RR}}{\partial q_{c}}=-c_{c}-\lambda_{1}+\lambda_{2}+\lambda_{4}=0$, $\lambda_{1}\left(q_{n}-q_{c}\right)=0$, $\lambda_{2}\left(q_{c}-q_{r}\right)=0$, $\lambda_{3}\left(q_{r}-\tau_{r}q_{n}\right)=0$, $\lambda_{4}\left(q_{c}-\tau_{c}q_{n}\right)=0$, $\lambda_{i}\geq 0,\;i=1,2,3,4$, $q_{n}>q_{c}$, $q_{c}>q_{r}$, $q_{r}>\tau_{r}q_{n}$ and $q_{c}>\tau_{c}q_{n}$, we get four solutions, among which, solutions 3–5 are the same as the corresponding ones under regulation only with collection target. After the redundant OCs are eliminated, we characterize the optimal solutions as follows:

Solution 1 (*RR^MM^*): Here, $q_{c}^{*}=\tau_{c}q_{n}^{*}$, $q_{r}^{*}=\tau_{r}q_{n}^{*}$, $\lambda_{1}^{*}=\lambda_{2}^{*}=0$, $\lambda_{4}^{*}=c_{c}$ and $\lambda_{3}^{*}=\frac{\delta \left(1+\tau_{r}\right)\left[\gamma_{1}-c_{c}\tau_{c}+\left(c_{c}+\gamma_{2}\right)\tau_{r}\right]}{1+\delta \tau_{r}\left(2+\tau_{r}\right)}-\left(\gamma_{2}+c_{c}\right)$. The optimality condition $\lambda_{3}^{*}\geq 0$ can be written as $u\leq t_{4}$.

Solution 2 (*RR^MV*^*): Here, $q_{c}^{*}=\tau_{c}q_{n}^{*}>q_{r}^{*}\text{>}\tau_{r}q_{n}^{*}$, $\lambda_{1}^{*}=\lambda_{2}^{*}=\lambda_{3}^{*}=0$, and $\lambda_{4}^{*}=c_{c}$. The optimality condition $q_{c}^{*}>q_{r}^{*}$ implies $u<t_{3}$, while $q_{r}^{*}\text{>}\tau_{r}q_{n}^{*}$ implies $u>t_{4}$, furthermore $t_{3}>t_{4}$ (as $\tau_{r}<\tau_{c}$). Note that this is the same as solution 1 under regulation only with collection target (*R^MV*^*), however, the lower boundary of *u* is different. □

**Proof of Corollary 3.** According to $u=\frac{c_{n}-c_{n}\delta +\left(1-\delta \right)\delta \tau_{r}-c_{c}\delta \tau_{c}\left(1+\tau_{r}\right)}{1+\delta \tau_{r}}$, $\gamma_{1}=1-c_{n}$ and $\gamma_{2}=\delta -c_{c}-c_{n}+u$, we have $\hat{\tau_{r}}$. □

**Proof of Proposition 4.** The Lagrange function is $L_{}^{NR}=\Pi^{NR}+\lambda_{1}\left(q_{n}-q_{c}\right)+\lambda_{2}\left(q_{c}-q_{r}\right)+\lambda_{3}q_{r}$, where $\lambda_{i}\geq 0,\;i=1,2,3$ are Lagrange multipliers. The corresponding Hessian Matrix is $H=\left[\begin{array}{ccc} -2 & -2\delta & 0\\ -2\delta & -2\delta & 0\\ 0 & 0 & 0 \end{array}\right]$, as $\Delta_{1}=-2<0$, $\Delta_{2}=4\delta (1-\delta )>0$, and |H|=0, the Hessian Matrix is negative semidefinite, and thus the first-order conditions guarantee optimality. Then, solving the Karush-Kuhn-Tucker (KKT) conditions, $\frac{\partial L_{}^{NR}}{\partial q_{n}}=\gamma_{1}-2q_{n}-2q_{r}\delta -e\varepsilon +\lambda_{1}=0$, $\frac{\partial L_{}^{NR}}{\partial q_{r}}=\gamma_{2}+c_{c}-2q_{n}\delta -2q_{r}\delta -e\alpha \varepsilon -\lambda_{2}+\lambda_{3}=0$, $\frac{\partial L_{}^{NR}}{\partial q_{c}}=-c_{c}-\lambda_{1}+\lambda_{2}=0$, $\lambda_{1}\left(q_{n}-q_{c}\right)=0$, $\lambda_{2}\left(q_{c}-q_{r}\right)=0$, $\lambda_{3}q_{r}=0$, $\lambda_{i}\geq 0,\;i=1,2,3$, $q_{n}-q_{c}\geq 0$, $q_{c}-q_{r}\geq 0$ and $q_{r}\geq 0$, we can get three solutions:

Solution 1: Here, $q_{n}^{*}=\frac{1}{2}\left(\gamma_{1}-e\varepsilon \right)$, $q_{c}^{*}=q_{r}^{*}=0$, $\lambda_{1}^{*}=0$, $\lambda_{2}^{*}=c_{c}$ and $\lambda_{3}^{*}=\gamma_{1}\delta -\gamma_{2}+e\left(\alpha -\delta \right)\varepsilon$, the manufacturer only produces new products, however, we only focused on the situation that new and remanufactured products coexist.

Solution 2 (*NR^VV^*): Here, $q_{n}^{*}>q_{c}^{*}=q_{r}^{*}\text{>}$, $\lambda_{1}^{*}=\lambda_{3}^{*}=0$ and $\lambda_{2}^{*}=c_{c}$. The optimality condition $q_{r}^{*}>0$ implies $u>g_{0}$, while $q_{r}^{*}<q_{n}^{*}$ implies $u<g_{1}$.

Solution 3 (*NR^AA^*): Here, $q_{n}^{*}=q_{c}^{*}=q_{r}^{*}\text{>}$, $\lambda_{1}^{*}=\frac{\gamma_{2}-2\gamma_{1}\delta +\gamma_{2}\delta +e\varepsilon \left[\left(2-\alpha \right)\delta -\alpha \right]}{1+3\delta }$, $\lambda_{3}^{*}=0$ and $\lambda_{2}^{*}=c_{c}-\gamma_{1}+e\varepsilon +\frac{\left(1+\delta \right)\left[\gamma_{1}+\gamma_{2}-e\left(1+\alpha \right)\varepsilon \right]}{1+3\delta }$. The optimality condition $\lambda_{1}^{*}\geq 0$ can be written as $u\geq g_{1}$. □

**Proof of Corollary 4.** According to $u>g_{0}$, we get $\frac{\gamma_{2}-\alpha e\varepsilon }{\gamma_{1}-e\varepsilon }>\delta$. □

**Proof of Proposition 5.** The Lagrange function is $L_{}^{R}=\Pi^{R}+\lambda_{1}\left(q_{n}-q_{c}\right)+\lambda_{2}\left(q_{c}-q_{r}\right)+\lambda_{3}q_{r}+\lambda_{4}\left(q_{c}-\tau_{c}q_{n}\right)$. The Hessian is negative semidefinite, and thus the first-order conditions guarantee optimality. Then, solving the Karush-Kuhn-Tucker (KKT) conditions, $\frac{\partial L_{}^{R}}{\partial q_{n}}=\gamma_{1}-2q_{n}-2q_{r}\delta -e\varepsilon +\lambda_{1}-\lambda_{4}\tau_{c}=0$, $\frac{\partial L_{}^{R}}{\partial q_{r}}=\gamma_{2}+c_{c}-2q_{n}\delta -2q_{r}\delta -e\alpha \varepsilon -\lambda_{2}+\lambda_{3}=0$, $\frac{\partial L_{}^{R}}{\partial q_{c}}=-c_{c}-\lambda_{1}+\lambda_{2}+\lambda_{4}=0$, $\lambda_{1}\left(q_{n}-q_{c}\right)=0$, $\lambda_{2}\left(q_{c}-q_{r}\right)=0$, $\lambda_{3}q_{r}=0$, $\lambda_{4}\left(q_{c}-\tau_{c}q_{n}\right)=0$, $\lambda_{i}\geq 0,\;i=1,2,3,4$, $q_{n}>q_{c}$, $q_{c}>q_{r}$, $q_{r}>0$ and $q_{c}>\tau_{c}q_{n}$, after eliminate the redundant solutions, we get five solutions:

Solution 1, $q_{r}^{*}=0$, we don’t consider this case, as we assume manufacturer participates in remanufacturing in absence of take-bake regulation.

Solution 2 (*R^MV*^*): Here, $q_{c}^{*}=\tau_{c}q_{n}^{*}>q_{r}^{*}\text{>}$, $\lambda_{1}^{*}=\lambda_{2}^{*}=\lambda_{3}^{*}=0$ and $\lambda_{4}^{*}=c_{c}$. The optimality condition $q_{c}^{*}>q_{r}^{*}$ implies $u<g_{3}$.

Solution 3 (*R^MV^*): Here, $q_{r}^{*}=q_{c}^{*}=\tau_{c}q_{n}^{*}$, and $\lambda_{1}^{*}=\lambda_{3}^{*}=0$, $\lambda_{2}^{*}=\gamma_{2}+c_{c}-e\alpha \varepsilon -\frac{\delta \left(1+\tau_{c}\right)\left[\gamma_{1}+\gamma_{2}\tau_{c}-e\varepsilon \left(1+\alpha \tau_{c}\right)\right]}{1+\delta \tau_{c}\left(2+\tau_{c}\right)}$ and $\lambda_{4}^{*}=\frac{\gamma_{1}\delta \left(1+\tau_{c}\right)-\gamma_{2}\left(1+\delta \tau_{c}\right)+e\varepsilon \left[\alpha -\delta -\left(1-\alpha \right)\delta \tau_{c}\right]}{1+\delta \tau_{c}\left(2+\tau_{c}\right)}$. The optimality condition $\lambda_{2}^{*}\geq 0$ can be written as $u\geq g_{3}$, while $\lambda_{4}^{*}\geq 0$ can be written as $u\leq g_{2}$, what’s more, $g_{2}>g_{3}$.

Solution 4 (*R^VV^*): Here, $q_{n}^{*}>q_{r}^{*}=q_{c}^{*}\text{>}\tau_{c}q_{n}^{*}$, $\lambda_{1}^{*}=\lambda_{3}^{*}=\lambda_{4}^{*}=0$ and $\lambda_{2}^{*}=c_{c}$. The optimality condition $q_{c}^{*}\text{>}\tau_{c}q_{n}^{*}$ implies $u>g_{2}$, while $q_{r}^{*}<q_{n}^{*}$ implies $u<g_{1}$, furthermore $g_{1}>g_{2}$ (as $\tau_{c}<1$). Note that this is the same as solution 2 under no regulation (*NR^VV^*).

Solution 5 (*R^AA^*): Here, $q_{r}^{*}=q_{c}^{*}=q_{n}^{*}$, $\lambda_{3}^{*}=\lambda_{4}^{*}=0$, $\lambda_{1}^{*}=\frac{\gamma_{2}-2\gamma_{1}\delta +\gamma_{2}\delta +e\varepsilon \left[\left(2-\alpha \right)\delta -\alpha \right]}{1+3\delta }$ and $\lambda_{2}^{*}=c_{c}-\gamma_{1}+e\varepsilon +\frac{\left(1+\delta \right)\left[\gamma_{1}+\gamma_{2}-e\left(1+\alpha \right)\varepsilon \right]}{1+3\delta }$. The optimality condition $\lambda_{1}^{*}\geq 0$ can be written as $u\geq g_{1}$. Note that this is the same as solution 3 under no regulation (i.e., *NR^AA^*). □

**Proof of Corollary 5.** According to $u=1+c_{c}-\delta -e\varepsilon +e\alpha \varepsilon -\frac{\left(1-\delta \right)\left(1-c_{n}-e\varepsilon \right)}{1+\delta \tau_{c}}$, we have $\tau_{c1}$. And according to $u=\frac{\alpha e\varepsilon \left(1+\delta \tau_{c}\right)}{1+\delta \tau_{c}}+\frac{c_{n}-c_{n}\delta -e\delta \varepsilon -e\delta \varepsilon \tau_{c}+\delta \tau_{c}\left(1-c_{c}-\delta -c_{c}\tau_{c}\right)}{1+\delta \tau_{c}}$, we have $\tau_{c2}$. □

**Proof of Proposition 6.** The Lagrange function is $L_{}^{RR}=\Pi^{RR}+\lambda_{1}\left(q_{n}-q_{c}\right)+\lambda_{2}\left(q_{c}-q_{r}\right)+\lambda_{3}\left(q_{r}-\tau_{r}q_{n}\right)+\lambda_{4}\left(q_{c}-\tau_{c}q_{n}\right)$. The Hessian is negative semidefinite, and thus the first-order conditions guarantee optimality. Then, solving the Karush-Kuhn-Tucker (KKT) conditions, $\frac{\partial L_{}^{RR}}{\partial q_{n}}=\gamma_{1}-2q_{n}-2q_{r}\delta -e\varepsilon +\lambda_{1}-\lambda_{3}\tau_{r}-\lambda_{4}\tau_{c}=0$, $\frac{\partial L_{}^{RR}}{\partial q_{r}}=\gamma_{2}+c_{c}-2\delta q_{n}-2\delta q_{r}-e\alpha \varepsilon -\lambda_{2}+\lambda_{3}=0$, $\frac{\partial L_{}^{RR}}{\partial q_{c}}=-c_{c}-\lambda_{1}+\lambda_{2}+\lambda_{4}=0$, $\lambda_{1}\left(q_{n}-q_{c}\right)=0$, $\lambda_{2}\left(q_{c}-q_{r}\right)=0$, $\lambda_{3}\left(q_{r}-\tau_{r}q_{n}\right)=0$, $\lambda_{4}\left(q_{c}-\tau_{c}q_{n}\right)=0$, $\lambda_{i}\geq 0,\;i=1,2,3,4$, $q_{n}>q_{c}$, $q_{c}>q_{r}$, $q_{r}>\tau_{r}q_{n}$ and $q_{c}>\tau_{c}q_{n}$, we get four solutions, among which, solutions 3–5 are the same as the corresponding ones under regulation with collection target al.one. After the redundant OCs are eliminated, we characterize the optimal solutions as follows:

Solution 1 (*RR^MM^*): Here, $q_{c}^{*}=\tau_{c}q_{n}^{*}$, $q_{r}^{*}=\tau_{r}q_{n}^{*}$, $\lambda_{1}^{*}=\lambda_{2}^{*}=0$, $\lambda_{4}^{*}=c_{c}$ and $\lambda_{3}^{*}=\frac{\delta \left(1+\tau_{r}\right)\left[\gamma_{1}-c_{c}\tau_{c}+\left(c_{c}+\gamma_{2}\right)\tau_{r}-e\left(\varepsilon +\alpha \varepsilon \tau_{r}\right)\right]}{1+\delta \tau_{r}\left(2+\tau_{r}\right)}-\left(\gamma_{2}+c_{c}-e\alpha \varepsilon \right)$. The optimality condition $\lambda_{3}^{*}\geq 0$ can be written as $u\leq g_{4}$.

Solution 2 (*RR^MV*^*): Here, $q_{c}^{*}=\tau_{c}q_{n}^{*}>q_{r}^{*}\text{>}\tau_{r}q_{n}^{*}$, $\lambda_{1}^{*}=\lambda_{2}^{*}=\lambda_{3}^{*}=0$ and $\lambda_{4}^{*}=c_{c}$. The optimality conditions $q_{c}^{*}>q_{r}^{*}$ implies $u<g_{3}$, while $q_{r}^{*}\text{>}\tau_{r}q_{n}^{*}$ implies $u>g_{4}$, furthermore $g_{3}>g_{4}$ (as $\tau_{r}<\tau_{c}$). Note that this is the same as solution 1 under regulation only with collection target (*R^MV*^*), however, the lower boundary of *u* is different.

**Proof of Corollary 6.** According to $u=\frac{\alpha e\varepsilon \left(1+\delta \tau_{r}\right)}{1+\delta \tau_{r}}+\frac{c_{n}-c_{n}\delta -e\delta \varepsilon +\left(1-\delta \right)\delta \tau_{r}-e\delta \varepsilon \tau_{r}-c_{c}\delta \tau_{c}\left(1+\tau_{r}\right)}{1+\delta \tau_{r}}$, $\gamma_{1}=1-c_{n}$ and $\gamma_{2}=\delta -c_{c}-c_{n}+u$, we have $\hat{\tau_{r}}$. □

**Proof of Lemma 1.** 

(i) According to Proposition 5, in solution R^MV*^, $\frac{\partial q_{n}{}^{*}}{\partial \tau_{c}}=-\frac{c_{c}}{2\left(1-\delta \right)}<0$ and $\frac{\partial q_{r}{}^{*}}{\partial \tau_{c}}=-\frac{c_{c}}{2\left(1-\delta \right)}>0$. In solution *R^MV^*, $\frac{\partial q_{n}{}^{*}}{\partial \tau_{c}}=-\frac{2\delta \left(1+\tau_{c}\right)q_{n}+\lambda_{4}}{2\left(1+2\delta \tau_{c}+\delta \tau_{c}^{2}\right)}<0$ and $\frac{\partial q_{r}^{*}}{\partial \tau_{c}}=\frac{\left(\gamma_{1}-e\varepsilon \right)\left(1-\delta \tau_{c}^{2}\right)+2\left(\gamma_{2}-\alpha e\varepsilon \right)\tau_{c}\left(1+\delta \tau_{c}\right)}{2\left(1+2\delta \tau_{c}+\delta \tau_{c}^{2}\right)}>0$.
(ii) According to Proposition 6, in solution *RR^MM^*, $\frac{\partial q_{n}{}^{*}}{\partial \tau_{r}}=-\frac{2\delta \left(1+\tau_{r}\right)q_{n}+\lambda_{3}}{2\left(1+2\delta \tau_{r}+\delta \tau_{r}^{2}\right)}<0$ and $\frac{\partial q_{r}{}^{*}}{\partial \tau_{r}}=\frac{2\left(1+\delta \tau_{r}\right)q_{n}-\lambda_{3}\tau_{r}}{2\left(1+2\delta \tau_{r}+\delta \tau_{r}^{2}\right)}>0$. □

**Proof of Proposition 7.** There are two solutions (*R^MV*^* and *R^MV^*) that characterize the optimal strategies of the manufacturer under regulation with binding collection target in Proposition 5. Under solution *R^MV*^*, $\frac{\partial E}{\partial \tau_{c}}=e\frac{\partial q_{n}{}^{*}}{\partial \tau_{c}}+\alpha e\frac{\partial q_{r}{}^{*}}{\partial \tau_{c}}=-\frac{ec_{c}}{2\left(1-\delta \right)}+\frac{\alpha ec_{c}}{2\left(1-\delta \right)}=\frac{ec_{c}\left(\alpha -1\right)}{2\left(1-\delta \right)}<0$. Therefore, if $q_{r}<\tau_{c}q_{n}$, then $\frac{\partial E^{*}}{\partial \tau_{c}}\leq 0$. Under solution *R^MV^*, $\frac{\partial E}{\partial \tau_{c}}=e\frac{\partial q_{n}{}^{*}}{\partial \tau_{c}}+\alpha e\frac{\partial q_{r}{}^{*}}{\partial \tau_{c}}=-e\frac{2\delta \left(1+\tau_{c}\right)q_{n}+\lambda_{4}}{2\left(1+2\delta \tau_{c}+\delta \tau_{c}^{2}\right)}+\alpha e\frac{2\left(1+\delta \tau_{c}\right)q_{n}-\lambda_{4}\tau_{c}}{2\left(1+2\delta \tau_{c}+\delta \tau_{c}^{2}\right)}$. Therefore, if $q_{r}=\tau_{c}q_{n}$ and $\alpha >\bar{\alpha }$, then $\frac{\partial E^{*}}{\partial \tau_{c}}>0$, where $\bar{\alpha }=\frac{2\delta \left(1+\tau_{c}\right)q_{n}+\lambda_{4}}{2\left(1+\delta \tau_{c}\right)q_{n}-\lambda_{4}\tau_{c}}$. □

**Proof of Proposition 8.** Proposition 6 implies that under regulation with additional remanufacturing target, the optimal strategies are the same as ones under regulation only with collection target, except solution *RR^MM^*. Under solution *RR^MM^*, $\frac{\partial E}{\partial \tau_{r}}=e\frac{\partial q_{n}{}^{*}}{\partial \tau_{r}}+\alpha e\frac{\partial q_{r}{}^{*}}{\partial \tau_{r}}=-e\frac{2\delta \left(1+\tau_{r}\right)q_{n}+\lambda_{3}}{2\left(1+2\delta \tau_{r}+\delta \tau_{r}^{2}\right)}+\alpha e\frac{2\left(1+\delta \tau_{r}\right)q_{n}-\lambda_{3}\tau_{r}}{2\left(1+2\delta \tau_{r}+\delta \tau_{r}^{2}\right)}$. Therefore, if $q_{r}=\tau_{r}q_{n}$ and $\alpha >\bar{\bar{\alpha }}$, then $\frac{\partial E^{*}}{\partial \tau_{r}}>0$, where $\bar{\bar{\alpha }}=\frac{2\delta \left(1+\tau_{r}\right)q_{n}+\lambda_{3}}{2\left(1+\delta \tau_{r}\right)q_{n}-\lambda_{3}\tau_{r}}$. □

**Proof of Proposition 9.** (i) Comparing the carbon emissions in Table 7 with the carbon emissions in Table 5, we can obtain the former with less carbon emissions; (ii) According to Table 5, we have $\frac{\partial E^{*}}{\partial \varepsilon }<0$. □
